# Energy landscape–engineered iontronics enable artificial thermoreceptors for augmented bioinspired thermosensation

**DOI:** 10.1126/sciadv.ady2547

**Published:** 2025-11-07

**Authors:** Fan Li, Hua Xue, Xiuzhu Lin, Juan Wang, Juan Li, Hongran Zhao, Tong Zhang

**Affiliations:** ^1^ State Key Laboratory of Integrated Optoelectronics, JLU Region, College of Electronic Science and Engineering, Jilin University, Changchun 130012, P. R. China.; ^2^ Ice and Snow Tourism Resorts Equipment and Intelligent Service Technology Ministry of Culture and Tourism Key Laboratory, Jilin University, Changchun 130012, P. R. China.; ^3^ School of Public Health, Jilin University, Changchun, 130012, P. R. China.

## Abstract

Biological thermoreception extends beyond mere temperature detection, capturing nuanced cues such as sunlight, wind chill, humidity, and object contact. However, artificial thermoreceptors rarely match the acuity and perceptual richness with their biological counterpart, leaving a critical gap in thermosensory functionality for human-machine integration. Here, we introduce a structurally programmed iontronic platform that leverages energy landscape engineering in a heterogeneous polymer electrolyte to establish a continuous distribution of potential wells hosting a continuum of ionic carrier states. This architecture enables semiconductor-like thermally activated conduction by ionic carriers, achieving detection of temperature variations as small as 8 mK. Building on this platform, we develop a biomimetic ionic skin that interprets airflow, humidity, solar irradiation, thermal conductivity variations, and evaporative cooling through thermal cues, mirroring the multifaceted thermoreception of biological skin. This work complements the existing research paradigm of iontronics by integrating solid-state physics principles. The artificial thermoreceptor thus narrows the gap between artificial and biological thermosensation, advancing human-machine integration and biohybrid systems.

## Teaser

Ultra-sensitive iontronic thermoreceptors enable artificial skin to mimic diverse thermosensory functions.

## INTRODUCTION

Thermal phenomena offer critical insights into physical and chemical processes, shaping how organisms interact with their surroundings (*1*–*3*). By perceiving subtle heat exchanges across the skin, biological systems derive a wealth of environmental cues—from the warmth of sunlight and chill of a breeze to the cool touch of metal (*4*, *5*). Replicating this delicate thermosensation in artificial devices is essential for bridging the sensory gap between machines and living organisms, catalyzing breakthroughs in biohybrid systems and human-machine cooperation (*6*–*9*). Central to this vision is the development of integrated flexible artificial thermoreceptors with millikelvin-level sensitivity, matching that of biological counterparts (*10*–*12*).

Biological thermoreceptors rely on heat-activated transient receptor potential ion channels for robust thermal responses (*3*), whereas high-sensitivity artificial sensors typically harness thermally excited electrons in semiconductors (e.g., ceramic oxides) (*13*, *14*). The stark disparity in charge carrier species and mechanical moduli across their respective transport media poses formidable challenges to developing artificial thermoreceptors and achieving seamless human-machine integration. Although flexible electronic alternatives—such as low-dimensional semiconductors (*15*, *16*), conjugated polymers (*17*, *18*), and conductive-filler-percolated polymer composites (*19*, *20*)—have emerged, their reliance on π-electron conduction or interfacial electron transport often yields diminished temperature responses or confines operation to around 5°C ranges.

Iontronics—the study of ionics in soft electronic systems—promises to bridge this fundamental gap in charge carriers between biological and mechanical systems, offering a transformative platform for biohybrid technologies (*21*, *22*). Yet, replicating the robust, thermally activated carrier dynamics of semiconductors or ion channels remains elusive, owing to the absence of intrinsic energy bands in ionic materials and the complexity of reproducing ion-channel gating synthetically. Current iontronic devices predominantly rely on non-Faradaic interfacial effects or chemical interactions to modulate carrier dynamics (*23*, *24*). By these means, state-of-the-art iontronic temperature sensors—based on pseudo-crosslinked block copolymers (*25*), deep eutectic gels (*26*), ion-electron junctions (*27*), or macrocyclic compound-modified nanochannels (*28*)—have reached sensitivities near 8 mK by introducing temperature-dependent ion transport barriers via specific chemical moieties. However, reliance on specialized functional groups constrains design flexibility and complicates multifunctional integration, underscoring the need for a more predictive, generalizable framework (*25*).

**Fig. 1.**
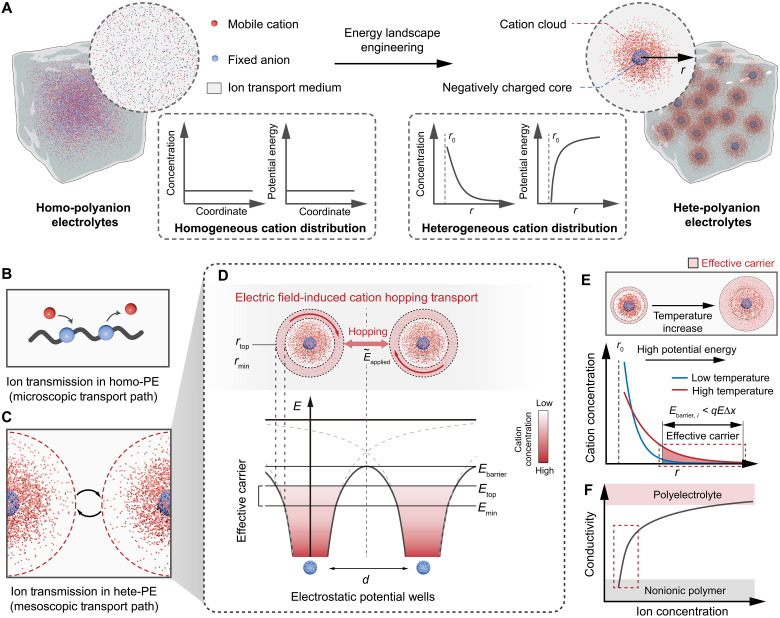
Structurally programmed ionic carrier dynamics in energy landscape engineered heterogeneous polymer electrolytes (hete-PE). (**A**) Comparison between a homogeneous polymer electrolyte (homo-PE) with uniformly distributed ions (left) and a hete-PE incorporating polyanionic submicron spheres that form localized cation clouds (right). Insets depict the respective cation concentration and potential energy profiles. (**B** and **C**) Schematic of ion transport pathways in homo-PE (B) versus hete-PE (C). (**D**) Energy landscape of hete-PE and the electric filed–induced cation hopping mechanism. (**E**) Schematic of temperature-driven redistribution of cations within the cation cloud, demonstrating how increasing temperature boosts the effective carrier concentration. (**F**) Schematic showing Kohlrausch-type relationship between ion concentration and polymer electrolyte conductivity, highlighting the rapid transition from insulating to conductive behavior at low carrier concentrations.

Drawing inspiration from solid-state models, we propose a conceptual shift from chemically gated to structurally programmed ionic transport by engineering the energy landscape in a heterogeneous polymer electrolyte (hete-PE) (Fig. 1A), thereby establishing a framework akin to semiconductor-like conduction principles. In our approach, dense fixed anions clusters within polyelectrolyte submicron spheres (PEMSs) create electrostatic potential wells, where thermal entropy drives mobile cations to diffuse against Coulomb forces. This arrangement forms a localized mesoscopic structure—a cation cloud surrounding each negatively charged core—where cations spontaneously occupy a continuum of electrostatic potential energy states akin to smoothly varying “energy levels.” Overlapping fields between adjacent cores define an ion-transport barrier dependent on their spacing. Consequently, ionic conduction proceeds via field-driven hopping of high-energy cations in the outer cloud layer. As temperature elevates these cations to higher energy states, the effective carrier concentration surges, enabling ultrahigh thermal responsiveness—reminiscent of thermally activated electrons in semiconductors. Meanwhile, thermal entropy governs the overall cation distribution, and core-to-core distance precisely tunes the transport barrier. Such a mesoscopic design provides a configurable energy landscape for optimized thermal modulation of carriers.

Building on this energy-landscape–engineered iontronic material, we demonstrate an artificial thermoreceptor capable of detecting temperature variations as small as 8 mK and achieving a sensitivity of ~1.2 mK. Integrating this sensor into a biomimetic framework yields a thermal-perception ionic skin that emulates the advanced thermosensory functions of biological skin. Beyond measuring absolute temperature, this ionic skin interprets airflow, humidity, thermal conductivity, evaporative cooling, and solar radiation under a unified temperature-sensation paradigm—promising transformative enhancements to artificial systems through human-like interpretation of subtle thermal cues. Crucially, by decoupling sensing performance from specific chemical interactions, our strategy offers a broadly applicable platform for high-performance iontronic sensors.

## RESULTS

### Design principle of energy landscape engineered hete-PE

To provide a well-defined and controllable platform without complications from chemically complex ionic species or polymer architectures, we developed an energy landscape-engineered hete-PE consisting of sodium poly(styrene sulfonate) (PSSNa) submicron spheres uniformly dispersed within a polyvinyl alcohol (PVA) matrix (figs. S1 to S5, and text S1). Upon dispersion, sodium cations dissociate from the sulfonate groups and become mobile charge carriers (text S2). Driven by entropy, these cations diffuse radially outward from each sphere, forming a spherical cation cloud around each negatively charged core (Fig. 1A). At equilibrium, electrostatic attraction counterbalances the cations’ tendency to spread out, producing a radial gradient in electric potential and cation density: The electric potential [ψ(*r*)] decays exponentially with radius (*r*) (multiplied by a 1/*r* geometric factor), while the cation density follows a Boltzmann distribution [fig. S6, A and B, and text S3, (i) to (ii)]

$$c\left(r\right)=c_{0}\text{exp}\left(-\frac{q\psi \left(r\right)}{k_{B}T}\right)$$ (1)

where *c*_0_ is a normalization constant (or far-field cation concentration), *q* is the cation charge, *k*_B_ is the Boltzmann constant, and *T* is the absolute temperature. Because of spherical symmetry, all cations at a given radius share the same potential energy, thereby creating a continuum of radial “energy levels.”

When these mesoscopic domains are distributed throughout the polymer, overlapping electric fields generate a nonuniform energy landscape. In contrast, homogeneous polymer electrolytes (homo-PEs) with uniformly distributed anions exhibit a uniform potential, allowing cations to move freely (Fig. 1B). In our hete-PE, however, a cation must surmount an energy barrier, *E*_barrier_, between adjacent domains (Fig. 1C). Only cations in the higher-energy outer shell meet the escape criterion under the applied electric field *E*_applied_ (Fig. 1D and text S3, (iii)]

$${\mathrm{qE}}_{\text{applied}}\Delta x>E_{\text{barrier},i}$$ (2)

where Δ*x* is the relevant hop distance, and *E*_barrier,*i*_ is the energy difference from the cation’s position at the saddle point *r*_min_ to the top of the barrier. Lower-energy cations remain bound near the negatively charged core and do not contribute to long-range transport.

By integrating the local Boltzmann distribution, one obtains an effective charge-carrier concentration *c*_eff_—the fraction of ions able to traverse interdomain barriers [text S3, (iv)]

$$c_{\text{eff}}=4\pi \int_{r_{\text{min}}}^{r_{\text{top}}}c\left(r\right)r^{2}dr$$ (3)

where *r*_top_ approximates the outer boundary of the ion cloud, *r*_min_ is a saddle point where the inward Coulomb attraction balances the outward field force. As temperature rises, more ions occupy higher-energy states, boosting *c*_eff_—thereby enhancing ionic conductivity (Fig. 1E and fig. S6C). Analogous to how heating a semiconductor raises electron occupancy in the conduction band, hete-PE’s temperature-driven cation redistribution notably magnifies conductivity when *c*_eff_ is initially low, which in agreement with Kohlrausch’s law of ionic conduction (Fig. 1F and fig. S7).

**Fig. 2.**
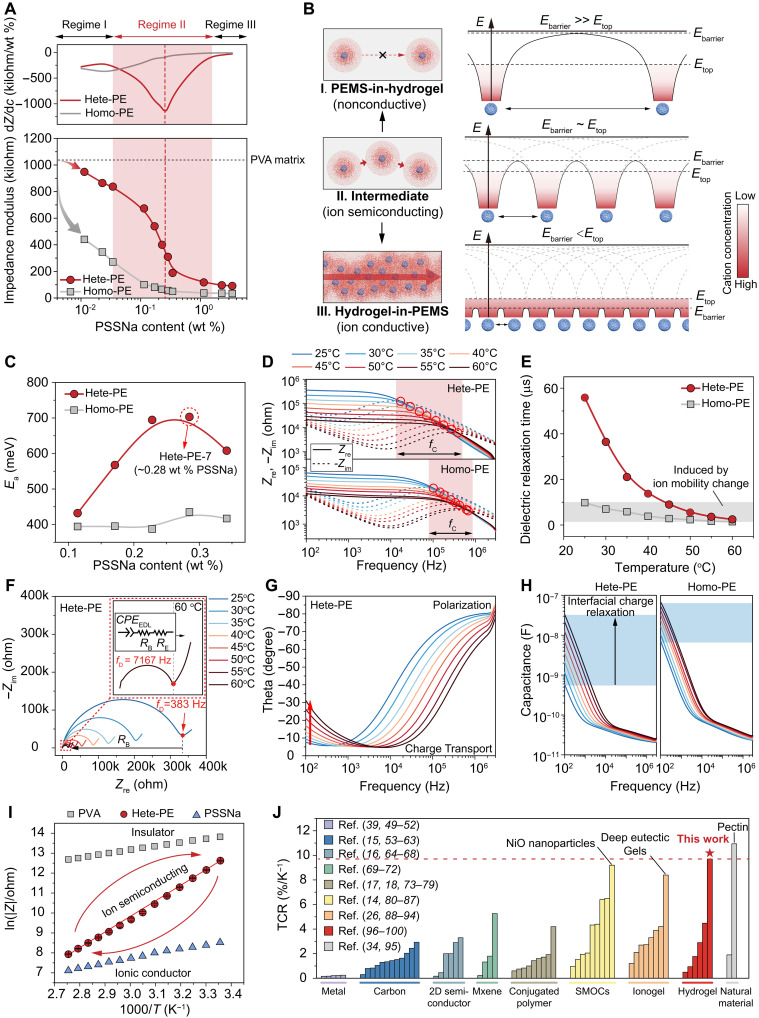
Conduction regimes and thermal activation of ionic transport in heterogeneous polymer electrolytes. (**A**) Impedance modulus of homo-PE (gray squares) and hete-PE (red circles) as a function of PSSNa loading, revealing three distinct conduction regimes in hete-PE. (**B**) Schematic illustrations of the energy landscape transition with PEMS spacing decrease: from nonconductive (“PEMS-in-Hydrogel”) to ion-semiconducting (intermediate) and finally ion-conductive (“Hydrogel-in-PEMS”). (**C**) Activation energies (*E*_a_) for homo-PE versus hete-PE as PSSNa loading increases. (**D**) Temperature-dependent real (*Z*_re_, solid lines) and imaginary (*Z*_im_, dashed lines) impedance spectra (25°–60°C) for homo-PE and hete-PE, with the crossover frequency (*f*_C_) indicated. (**E**) Comparison of variation tendencies of dielectric relaxation times of homo-PE and hete-PE as a function of temperature. (**F** and **G**) Representative Nyquist plots (F) and phase-angle plots (G) of hete-PE at varied temperatures, transitioning from charge transport dominant to polarization dominant regimes at low frequency. (**H**) Comparison of variation tendencies of frequency-dependent capacitance for homo-PE and hete-PE at different temperature. (**I**) Arrhenius plots contrasting PVA (insulator), hete-PE, and PSSNa (ionic conductor). (**J**) Comparison of temperature coefficient of resistance (TCR) values of this work with other state-of-the-art thermistor materials (*14*–*18*, *26*, *34*, *39*, *49*–*100*).

Because overlapping potential wells can reduce *E*_barrier_, adjusting the domain-to-domain spacing (via PSSNa sphere concentration) tunes the barrier height. Accordingly, the hete-PE exhibits a three-regime conductivity transition (1 kHz, 1 V, 25°C) with PSSNa weight percent rising (detail in table S1 and text S1), unlike the monotonic response in homo-PE (Fig. 2A). At low PSSNa content (regime I), wells barely overlap, forming large barriers; the material behaves almost similar to pure PVA (Fig. 2, B and I). Conversely, at high PSSNa content (regime III), neighboring wells merge into a quasi-continuous potential, yielding a homogeneous electrolyte where ions transport freely (Fig. 2B, III). In the intermediate regime (regime II), a sharp shift from insulating to conductive behavior emerges around ~0.28 weight % (wt %) PSSNa, marking a percolation-like threshold. Here, the activation energy (*E*_a_) peaks (Fig. 2C; figs. S6, D to I and S8; and text S4), confirming that ion transport barriers depend on domain spacing (*29*, *30*) and that electric field–induced cation hopping transport is facilitated by overlapping potential fields. In contrast, the impedance change rate and the activation energy of homo-PE remain low and nearly constant across all PSSNa concentrations, indicating that ion transport in the homogeneous system is relatively unimpeded.

The distinctive energy landscape and ion-transport mechanism in hete-PE engender electrical behavior fundamentally different from that of conventional homo-PE. Notably, the interdomain transport barrier, spanning three structural regimes—“PEMS-in-hydrogel” (regime I), intermediate (regime II), and “hydrogel-in-PEMS” (regime III)—parallels how bandgap widths distinguish insulators, semiconductors, and conductors. This analogy situates intermediate hete-PE as a soft-matter platform that mirrors thermally modulated conductivity transitions seen in classical semiconductor systems. For clarity of analysis, our energy landscape model focuses on the electrostatic potential generated by negatively charged cores. Secondary effects—such as local chemical potential variations arising from hydroxyl groups or from the nonuniform distribution of water molecules—are treated as background potentials superimposed upon the electrostatic potential. Given their comparatively minor influence, these factors are assumed not to affect the essential features of the model (see text S5).

Complex impedance spectroscopy (CIS) provides crucial insights into these dynamics (text S6). We analyzed Nyquist and Bode plots of hete-PE (~0.28 wt % of PSSNa) at various temperatures to examine how thermal effects modulate ion behavior. In Bode plots, the intersection of real (*Z*_re_) and imaginary (*Z*_im_) impedance traces marks the charge relaxation frequency (*f*_C_)—the highest frequency at which ionic carriers can track an alternating field. As either carrier concentration or mobility rises, *f*_C_ shifts to higher frequencies (*31*). In homo-PE, shift in *f*_C_ primarily reflects temperature-induced changes in ionic carrier mobility. By contrast, hete-PE exhibits a far more pronounced *f*_C_ shift (25° to 60°C), indicating a strong thermal modulation of effective carrier concentration (Fig. 2D). Consequently, the charge relaxation time [τ = 1/(2π*f*_C_)] in hete-PE decreases more steeply with temperature (Fig. 2E), analogous to the effect of artificially raising ion concentration in homo-PE (fig. S9). This contrast underscores that thermally driven redistribution of cations into higher-energy states notably boosts the effective carrier density in hete-PE.

Nyquist plots further corroborate this thermal modulation. The semicircle diameter of hete-PE (~0.28 wt % of PSSNa) contracts noticeably at higher temperatures, indicating a pronounced reduction in bulk resistance (*R*_B_) (Fig. 2F). Concurrently, the low-frequency tail becomes more prominent, and the Bode phase angle shifts from 0° to below −30°, signaling a shift from charge transport-dominated dynamics to interface polarization-dominated behavior, indicative of electric double layer (EDL) formation (Fig. 2G) (*32*). Because the EDL capacitance (*C*_EDL_) scales with $\sqrt{n}$ in dilute electrolytes (where *n* is the carrier concentration), this temperature-driven increase in low-frequency capacitance directly reflects a higher effective carrier density (Fig. 2H) (*33*). Notably, at 25°C, the Bode and Nyquist plots of hete-PE (~0.28 wt % of PSSNa) resemble those of homo-PE containing just ~0.01 wt % PSSNa, whereas at 60°C, they closely match homo-PE at ~0.28 wt % of PSSNa (figs. S11 and S12). In contrast, the homo-PE with 0.01 wt % of PSSNa shows little temperature dependence (fig. S13).

Because carrier concentration in hete-PE depends strongly on temperature, its conductivity transitions continuously from nonionic polymer-like behavior to that of a homo-PE–type ionic conductor over 25° to 90°C (Fig. 2I). In the ambiently relevant conditions (25° to 60°C), hete-PE achieves a temperature coefficient of resistance (TCR) of TCR = −9.7% at 25°C and thermal index *B* = 8613 K, surpassing both homo-PE and nonionic polymers (Fig. 2J and table S2; see text S4 for definitions). Intriguingly, its TCR also exceeds classic high-TCR metal-oxide thermistors (*13*, *14*), trailing only pectin (*34*, *35*)—a polysaccharide celebrated for its extreme thermal responsivity. (Note that this comparison excludes switch-type thermosensitive materials such as conductive filler-percolated composites (*19*, *20*, *36*) and phase-transition ionic conductors (*37*, *38*), which exhibit high TCRs but are limited to narrow temperature ranges.)

By implementing structurally programmed ionic transport rather than relying on chemical functionalization, we reduce dependence on specialized chemical moieties while preserving robust thermal responsiveness. Consequently, additional functionalities—such as the self-healing hydrogen-bond network formed by cellulose nanofibers (CNFs) (fig. S14)—can be introduced without diminishing the TCR. Although the PVA/CNF composite differs markedly from neat PVA in its mechanical properties, the resulting hete-PE still delivers a TCR of approximately −8.7%. This preservation of thermal responsiveness despite compositional changes suggests that structurally programmed ionic transport holds promise for extension across diverse hydrogel and ionogel systems, where stability, operational range, biocompatibility, and environmental resilience can be tailored for specific application demands.

### Characterization of temperature sensing properties

**Fig. 3.**
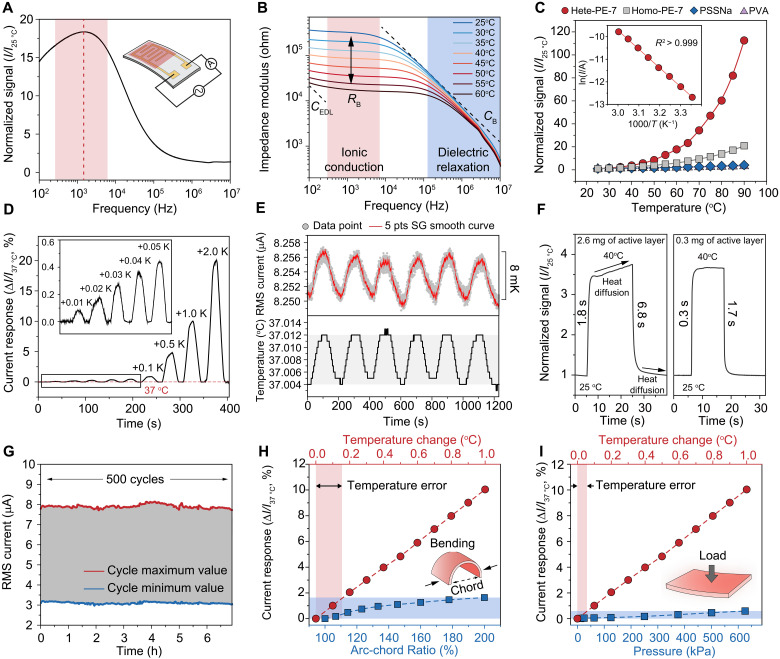
High-sensitivity thermoresponse of the flexible hete-PE sensor and its mechanical robustness. (**A**) Frequency-dependent normalized current response (25°C–60°C, 1 V) showing a peak at ~1.4 kHz for maximum signal; inset illustrates the measurement setup. (**B**) Bode plots of hete-PE at various temperatures. (**C**) Comparison of normalized current signals among sensors based on PVA, PSSNa, homo-PE, and hete-PE. Inset: Arrhenius-like behavior (ln*I* versus 1000/*T*) for the hete-PE sensor with an excellent linear fit (*R*^2^ > 0.999). (**D**) Real-time current response to small temperature steps (Δ*T* = 0.01 to 2.00 K); *I*_0_ is the sensor current at 37°C. (**E**) Time evolution of RMS current (red) of hete-PE sensor under repeated faint heating-cooling cycles (Δ*T* ≈ 8 mK), with a commercial high-precision RTD (black) recorded simultaneously for calibration. (**F**) Dynamic response and recovery for sensors with different active-layer masses and thicknesses (2.6 mg, 130 μm versus 0.3 mg, 15 μm). (**G**) Long-term stability under 500 consecutive heating-cooling cycles, maintaining stable RMS current at 25°C and 40°C. (**H** and **I**) Influence of bending (H) and compression (I) on temperature reading; bending degree is quantified by arc-chord ratio, and pressure is varied up to 600 kPa.

To evaluate the temperature sensing performances of the hete-PE, we fabricated flexible sensor devices by drop-casting a hete-PE-7 (~0.28 wt % of PSSNa) film onto a plastic substrate patterned with gold interdigitated electrodes and encapsulating it within a thin polytetrafluoroethylene (PTFE) membrane (fig. S1). An AC voltage (1-V amplitude) was applied, and the root mean square (RMS) current response was measured under temperature variations. Initial tests revealed a strong dependence on excitation frequency (Fig. 3A). Within 100 Hz to 10 MHz, we identified 1.4 kHz as the optimal operating frequency, where ionic transport dominates impedance and thus maximizes the thermal response (Fig. 3B and fig. S15). Consequently, 1 kHz was adopted for subsequent measurements, following standard iontronic sensing practices.

Because hete-PE exhibits thermally tunable carrier concentration, its sensor response increases by over 130-fold between 25° and 90°C (Fig. 3C)—far exceeding typical polymer electrolytes, where temperature sensing is restricted by ionic mobility alone. Although the current-temperature profile is nonlinear, it follows an Arrhenius-like trend characteristic of thermally activated ion hopping in the heterogeneous energy landscape. A plot of ln *I* versus 1/*T* (25° to 60°C) yields a near-perfect linear fit (*R*^2^ > 0.999) (Fig. 3C, inset).

Achieving biological-grade thermoreception demands millikelvin sensitivity near physiologically relevant temperatures. Evaluating the sensor at ~37°C, we estimated a theoretical detection limit of ~1.2 mK from baseline noise (fig. S16) (*25*, *39*). The device resolved consecutive thermal steps from 37° to 39°C, producing a 10% current change per 1.0°C step (Fig. 3D) and consistently distinguished temperature differentials as small as 8 mK (Fig. 3E)—the highest reported measured sensitivity for any resistive-type flexible temperature sensor to date. This performance surpasses the precision of human thermoreceptors (*10*), demonstrating millikelvin-level sensitivity.

Beyond sensitivity, the sensor shows rapid response and negligible thermal hysteresis (fig. S17). As heat diffusion through the active layer is the rate-limiting step of thermal response, decreasing the thermal mass shortens the response time. Reducing the film mass to ~0.3 mg (thickness ~15 μm) shortened the response time to ~0.3 s and the recovery to ~1.8 s (Fig. 3F). Further structural miniaturization and enhancement of thermal conductivity could improve these time constants. For comparison, table S2 summarizes the performances of state-of-art flexible temperature sensors. Among iontronic devices, our hete-PE sensor achieves both the highest sensitivity and the fastest response/recovery times reported thus far, positioning it as a promising platform for artificial thermoreceptors.

### Specificity and stability of thermal sensing

In addition to its exceptional temperature sensing performances, an artificial thermoreceptor must demonstrate durability and temperature specificity for real-world viability (*40*, *41*). We thus examined the hete-PE sensor’s stability and mechanical robustness under various conditions. In cyclic tests, the device underwent 500 repeated temperature cycles (25° to 40°C) with minimal drift, maintaining an error under ±0.15°C (Fig. 3G). Over 20 days of continuous monitoring, no appreciable change in impedance was observed at any temperature (fig. S18), confirming excellent long-term reliability.

Next, we evaluated mechanical durability. Under bending to half of its original span—a substantial curvature for a thin-film sensor—the temperature reading deviated by less than 0.15°C (Fig. 3H). Compression tests up to 600 kPa (exceeding typical wearable-sensor pressures) introduced an error below 0.05°C (Fig. 3I). Even after 2000 bending cycles, the thermal response decreased by under 0.6% (fig. S19), highlighting the device’s robustness for biohybrid or soft-robotic applications.

We also tested the sensor’s environmental specificity by varying ambient humidity, which can affect iontronic materials via enhanced polymer chain mobility—akin to the effect of temperature. An unencapsulated hete-PE device showed notable impedance shifts under fluctuating humidity. However, encapsulating it with a PTFE “waterproof” membrane stabilized impedance across a wide humidity range (fig. S20). This demonstrates that, with proper encapsulation, the hete-PE thermoreceptor remains immune to humidity-induced interference, sustaining reliable temperature-driven signals.

### Biomimetic thermal perception ionic skin

**Fig. 4.**
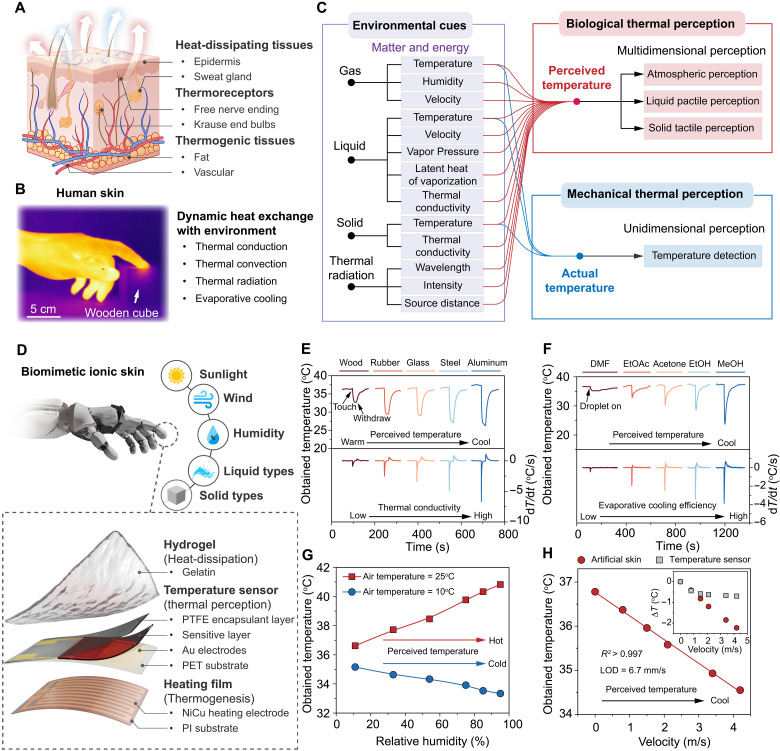
Biomimetic ionic skin replicating the dynamic thermoreception of biological skin. (**A**) Schematic of the human thermosensory system. (**B**) Infrared image illustrating various heat exchange modes at the skin–environment interface. (**C**) Comparison of environmental cues perceived by biological thermoreception versus conventional mechanical systems. (**D**) Structural schematic of the biomimetic ionic skin. (**E** and **F**) Obtained temperature (top) and derivative curves (bottom) when the ionic skin contacts solids of different thermal conductivities (E) and droplets of various liquids (F). (**G**) Humidity-dependent obtained temperature at ambient temperatures of 10° and 25°C, replicating the damp-cold versus sultry-warm sensations in real skin. (**H**) Relationship between airflow velocity and the obtained temperature (LOD ~6.7 mm/s). Inset: Comparison of airflow-induced cooling in the ionic skin (red) versus a direct temperature sensor (gray). LOD, limit of detection.

Conventional artificial temperature sensors typically measure absolute temperature by reaching thermal equilibrium with a target, which halts net heat exchange. In contrast, biological organisms experience perceived temperature—a subjective sensation integrating additional factors such as ambient humidity and convective airflow (*42*). This richer thermosensation arises from the skin’s thermoregulatory system, which balances internal heat generation and dissipation (Fig. 4A). Through dynamic feedback, warm-blooded organisms maintain core temperatures above ambient while monitoring conduction, convection, radiation, and evaporation (Fig. 4, B and C) (*43*). As a result, identical ambient temperatures can feel markedly different—metals often feel cooler than wood, and humid air can amplify either stifling heat or penetrating cold.

To emulate this complexity, we constructed a biomimetic ionic skin (Fig. 4D) that uses our hete-PE sensor as the artificial thermoreceptor. A gelatin-based hydrogel layer sits atop the sensor, leveraging its water retention, interfacial adhesion, and conformability to form a tightly integrated heat-dissipating layer (*44*). Beneath the sensor, a polyimide (PI) heating film sustains an internal temperature near 37°C—mimicking the warm-blooded condition. In operation, the ionic skin measures its own “obtained temperature” in response to various external stimuli, such as airflow, moisture, radiant heat, or contact with solids and liquids.

When the ionic skin contacts solid objects of different thermal conductivities (e.g., metal versus wood), it registers distinct obtained temperatures (Fig. 4E). Higher-conductivity materials draw heat more rapidly, prompting faster cooling and a lower steady-state temperature, mirroring the sensation of cold metal on human skin. Time-dependent analysis revealed that the initial rate of temperature changes upon contact showed a linear relationship with the logarithm of the material’s thermal conductivity (*R*^2^ > 0.98) (figs. S21 and S22, details of solid thermal conductivity see table S3). Similarly, placing droplets (30 μl) of different liquids (e.g., methanol or water) on the surface induces temperature shifts correlated to their vapor pressure, heat of vaporization, and latent heat draw (table S4) (*45*). By analyzing peak cooling rates, the system can differentiate high-purity laboratory solvents (Fig. 4F) and even gauge the volume ratio of the two-component solvent mixture (fig. S23A), a capability that conventional sensors, reading only absolute temperature, cannot match (figs. S23B and S24).

To capture additional environmental cues, the gelatin layer provides an evaporative interface sensitive to ambient humidity, wind speed, and irradiance. At 10°C, rising humidity obviously lowers the ionic skin’s obtained temperature by enhancing conductive heat loss, whereas at 25°C, the same increase in moisture raises the obtained temperature due to the blocked evaporative cooling (Fig. 4G)—effects paralleling human thermal perception. Moreover, a near-linear dependence of obtained temperature on humidity suggests that the ionic skin can gauge humidity within a practical range, in contrast to conventional sensors that show negligible response (fig. S25B). Removing the gelatin layer markedly weakens this humidity sensitivity, with the measured temperature dropping consistently as humidity increases (fig. S25C)—highlighting the critical role of the hydrogel-based evaporative interface. Under controlled airflow, the obtained temperature decreases proportionally to wind speed (*R*^2^ > 0.99), modeling a “wind chill” phenomenon with a limit of detection (LOD) of about 6.7 mm/s (Fig. 4H). In contrast, a standalone hete-PE sensor exhibits minimal airflow sensitivity, with its response saturating above 1 m/s (fig. S26B). Notably, removing the gelatin layer leaves only the heater and sensor in a configuration analogous to classic airflow velocity detectors (*13*), thereby improving low-speed sensitivity to 0.13 mm/s. However, without the gelatin layer, the airflow-temperature relationship becomes nonlinear and diverges from biologically representative behavior (fig. S26, C and D). Under incandescent illumination, the obtained temperature rises according to lamp intensity, introducing the dark dye in the hydrogel that can further enhance this absorption (fig. S27), akin to feeling sunlight warms the skin. This approach enables artificial skins to detect radiative heat sources or solar intensity directly.

**Fig. 5.**
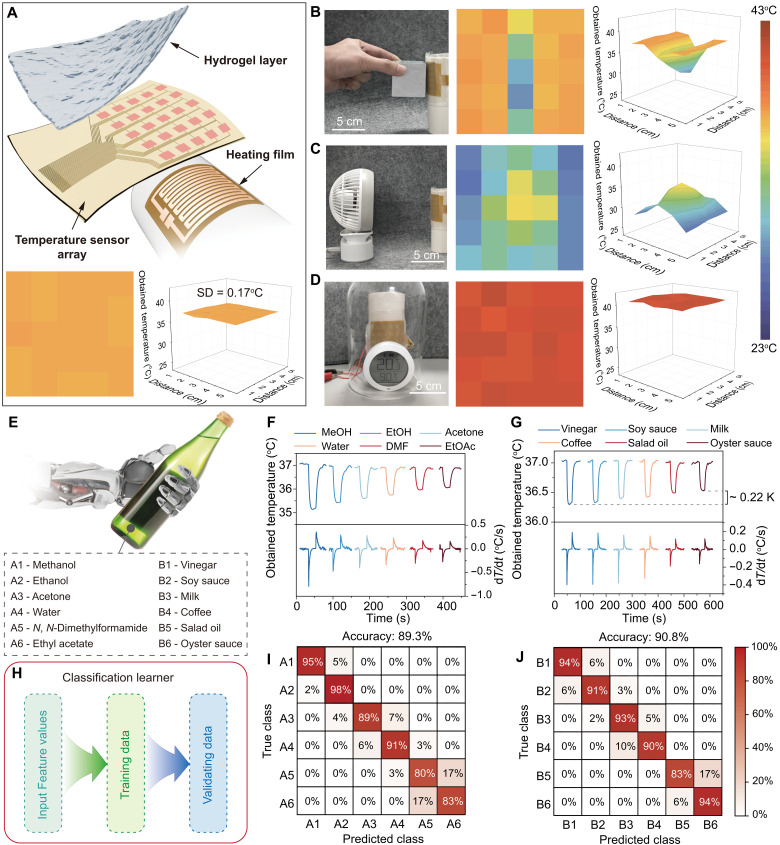
Spatial temperature mapping and noninvasive liquid identification. (**A**) Schematic of the biomimetic ionic skin featuring a 5 by 5 pixel array of artificial thermoreceptors (top), and the corresponding baseline temperature map at ambient conditions (bottom). (**B** to **D**) Representative two-dimensional (2D) (middle) and 3D (right) temperature maps of the ionic-skin array under three scenarios (left): contact with a room-temperature metal (B), exposure to unidirectional airflow (6.4 m/s) (C), and increased ambient humidity (D). (**E**) Set of 12 liquids for classification, comprising six high-purity laboratory solvents and six common food/beverage items. (**F** and **G**) Measured temperature curves (top) and corresponding derivatives (bottom) when the ionic skin contacts bottles containing distinct solvents (F) and edible liquids (G). (**H**) Workflow of the classification learner used to identify each liquid. (**I** and **J**) Confusion matrices demonstrating classification accuracies of 89.3 and 90.8%, respectively, for the two liquid sets.

Environmental stimuli often induce comparable temperature changes at a single thermoreceptor, yet biological systems differentiate among them by deploying arrays of thermoreceptors for spatial resolution. Following this example, we incorporated a 5 by 5 pixel array of artificial thermoreceptors into our biomimetic ionic skin (Fig. 5A and fig. S28) and mounted it onto a curved surface to mimic a limb or body segment. By mapping each pixel’s obtained temperature, we generated two-dimensional “thermal images” under three representative conditions: (i) contact with a room-temperature metal block, (ii) exposure to unidirectional airflow, and (iii) an increase in ambient humidity. Each scenario produced distinctly different thermal patterns.

When a metal cube was pressed onto the skin array, only those pixels directly in contact displayed a marked drop in temperature, delineating a sharply defined cool region with steep lateral gradients (Fig. 5B), and the thermal image well matched the geometry of the contact area (fig. S29, C and D). By contrast, airflow induced a broader zone of cooling devoid of clear boundaries (Fig. 5C and fig. S29, E and F). From a fluid-dynamics perspective, air flowing around a cylinder encounters a stagnation point at the front (highest pressure, lowest velocity) and then accelerates as it moves along the curved surface—leading to temperature contours that mirror the predicted velocity distribution. A notable rise in humidity instead caused a nearly uniform temperature shift across the array (Fig. 5D and fig. S29, G and H). By comparing these spatial patterns, the array-based system effectively discerns not only a cold solid object from a cold breeze but also other subtle environmental differences. In this way, the ionic skin achieves a more human-like thermosensory capacity, offering promise for robotics or prosthetic devices that demand nuanced environmental awareness. Further integration with vision or tactile sensors could provide even richer multimodal perception for artificial systems (*46*, *47*).

Last, leveraging the ultrahigh sensitivity of our artificial thermoreceptor—exceeding even human thermal discrimination—we explored a capability beyond typical human capability: identifying liquid contents in sealed glass bottles by “thermal touch” (Fig. 5E). We prepared 12 identical glass bottles, each containing a distinct liquid (six high-purity solvents and six common beverages/condiments), all initially at room temperature. The biomimetic ionic skin contacted each container 100 times, recording transient temperature profiles to capture heat exchange. As an index for classification, we extracted the peak cooling rate—the maximum derivative of the obtained temperature curve (Fig. 5, F to H). A linear support vector machine (SVM) with fivefold cross-validation then distinguished among the 12 liquids with ~89.3% accuracy for the solvents and ~90.8% for edible liquids (Fig. 5, I and J). Notably, some liquids were thermally similar—soy sauce, vinegar, coffee, and milk, for example, are all water-based and at the same temperature, yet the ionic skin consistently differentiated them, even when their obtained-temperature difference was only ~26 mK. This discrimination surpasses both human and conventional sensor capabilities, as the latter often rely on additional sensory (e.g., visual) inputs for comparable accuracy (*48*). This precise thermal recognition can facilitate noninvasive material identification, quality control, and even search-and-rescue scenarios—expanding the practical horizons of artificial thermosensory technology.

## DISCUSSION

In summary, we present a unified materials-and-device strategy that integrates a fundamental iontronic mechanism with bioinspired sensing functionality. By engineering the ionic energy landscape in a soft polymer electrolyte, we developed a flexible iontronic material that mimics semiconductor physics using ions—constructing continuous energy levels for ionic carriers and enabling thermal migration between these levels to generate effective charge carriers. This energy landscape-engineered iontronics provides remarkable thermal sensitivity in a flexible sensor, capable of detecting millikelvin-scale temperature changes. Leveraging this material, we achieved an artificial thermoreceptor with sensitivity and response speed surpassing any existing flexible iontronic sensor, approaching the performance of biological counterparts.

Beyond the material innovation, we demonstrated how this artificial thermoreceptor can be integrated into a biomimetic thermal perception ionic skin, imparting machines with skin-like sensing capabilities. Our system can interpret complex thermal stimuli—ranging from humidity and wind to direct contact and radiation—producing perceptual outputs akin to human sensations. This represents an important step toward artificial systems that not only measure the environment but also experience it in a more human-like manner. These capabilities are particularly relevant to robotics and prosthetic skins, where the ability to interpret subtle environmental cues and respond safely is crucial. For instance, a robot equipped with this thermal perception skin could detect a breeze or a damp surface and react as a human would, enhancing safety and adaptability in human-robot interactions. In addition, it expands the sensory dimension of artificial skin without requiring complex sensor arrays using thermal sensing as a universal cue to infer multiple environmental parameters.

At a broader level, this study provides a complementary perspective within iontronics and materials science. The concept of designing mesoscale charge landscapes to control ionic transport can be extended to other ions, ionic mixtures, or even hybrid devices combining ionic and electronic conduction. By precisely arranging charged domains, we could emulate solid-state phenomena—such as p-n junctions, energy filters, or neuron-like thresholds in ionic circuits—bridging the gap between classical electronic devices and ionic systems. This energy landscape engineering approach propels iontronics distinct from chemistry-driven effects, providing a physics-based framework that complements existing strategies such as interfacial polarization and ion-molecule interactions. Ultimately, our findings deepen the understanding of ionic charge carrier dynamics and demonstrate how harnessing these principles can drive sensory advancements, bringing artificial devices closer to the rich perceptual world of living organisms.

Although energy landscape–engineered iontronics has demonstrated considerable promise, several challenges remain. For simplicity, our model mainly considered the contribution of electrostatic potentials to the energy landscape, while additional factors—such as functional groups in the polymer matrix and the heterogeneous distribution of water molecules—may generate chemical potential gradients that were not explicitly analyzed. The nature of ionic carriers is also critical: variations in charge number, size, or chemical properties could reshape the energy landscape and alter transport characteristics, warranting systematic investigation. It will therefore be valuable to complement experimental studies with molecular dynamics simulations to further clarify the underlying transport mechanisms and their role in thermal sensing.

At the device level, our work focused primarily on enhancing thermal response, so that subtle signals could be distinguished under interfering stimuli. However, further structural optimization is required to decouple deformation from thermal response and thereby improve sensing precision. Within the ionic skin system, water content fluctuations in the hydrogel dissipation layer are a common challenge for hydrogel-based devices and inevitably affect evaporation-driven heat dissipation. While these fluctuations may lead to baseline drift, the functional responses of our ionic skin were preserved by adjusting heater power to re-establish equilibrium. This design thus involves a trade-off, sacrificing part of absolute precision to capture richer environmental information. Future robustness may be improved by adaptive heater control based on hydrogel conductivity, high-retention hydrogel formulations, and evaporation-regulating structures to achieve more reliable dehydration control and ensure long-term stability for practical applications.

## MATERIALS AND METHODS

### Synthesis of the PEMS

As outlined in fig. S1A, 8.7-mmol vinyltrimethoxysilane (VTMS) was dissolved in 100 ml of deionized (DI) water and stirred at 25°C for 30 min. Ammonia was then added dropwise to the until the pH reached 9.0, and the mixture was stirred continuously at 60°C for 4 hours. When the reaction was complete, a white suspension formed. The resulting precipitate was collected by centrifugation (11,000 rpm), washed with DI water, and dried in an oven at 60°C. Next, 870 mg of sodium vinylsulfonate was added to 5 ml of *N*-methyl pyrrolidone and stirred at 1000 rpm. The dried precipitate (100 mg) was dispersed in this solution and stirred for 10 min. Subsequently, 27.4 mg of azodiisobutyrodinitrile was introduced, and the mixture was stirred at 60°C for 48 hours. Afterward, it was transferred into excess diethyl ether to remove any unreacted solvents. The resulting white product was isolated by filtration, washed several times with methanol, and ultimately dried in an oven at 60°C for 8 hours to yield PEMS.

### Preparation of hete-PE precursor solution

To prepare the PVA precursor solution, 1.0 g of PVA and 0.3 g of glycerin were added to 8.7 ml of DI water and then stirred at 90°C for 4 hours. Next, different amounts of PEMS were dispersed into the PVA solution and continuously stirred for 1 hour, yielding the hete-PE precursor solutions. Hete-PE samples containing 0.2, 0.4, 0.6, 2.0, 3.0, 4.0, 5.0, 6.0, 20, 40, or 60 mg of PEMS were labeled as hete-PE-1 through hete-PE-11, respectively.

As a control, we prepared a homo-PE solution by adding PSSNa to the PVA hydrogel, ensuring that the mass fraction of PSSNa matched the calculated value in each corresponding hete-PE sample. These control samples were designated as homo-PE-1 to homo-PE-11.

### Fabrication of hete-PE sensors

Pre-cleaned poly(ethylene terephthalate) substrates (2 cm by 1 cm) were used, each bearing 20 pairs of interdigitated gold electrodes (line width and spacing both ≈ 50 μm). To fabricate the hete-PE sensors, 200 μl of the hete-PE precursor solution was drop-casted onto each substrate and allowed to dry under ambient conditions (25°C, 33% relative humidity) for 10 hours. PTFE tape served as the encapsulation layer (fig. S1, B and C). By the same procedure, homo-PE sensors were also prepared for comparison.

### Measurements

Electrical properties were characterized on a Keysight E4990A impedance analyzer (Keysight Technologies Inc., America) with a 1-V sinusoidal input (no dc bias) more than 20 Hz to 20 MHz. Sensor sensitivity was defined as the SD of the measured output at a constant temperature, achieved by placing the sensor in a thermally insulated enclosure. The normalized current was defined as *I*/*I*_25°C_, where the current at 25°C served as the baseline in laboratory evaluations. The sensor response was calculated as Δ*I*/*I*_37°C_, where the current at 37°C was used as the physiological baseline for ionic skin applications. Response and recovery times were determined by measuring the interval for a 90% change in RMS current during heating and cooling, respectively. Accuracy was taken as the deviation between sensor readings and a calibrated temperature measured by a Pt100 platinum resistor (YET-720 L, KAIPUSEN, China) with a sensitivity of better than 10 mK. A custom-built temperature control stage—comprising a 4 cm–by–4 cm alumina ceramic heating pad, a Peltier module mounted on a finned aluminum heat sink, and a programmable dc power supply (UTP 8303, UNI-T, China)—was used to generate and regulate subtle thermal shifts. In this configuration, the ceramic heater provided stable background heating, while the Peltier element, tuned by fine voltage steps, enabled precise cooling. Real-time monitoring and calibration with the commercial reference sensor (10-mK resolution) ensured accuracy. A schematic of the setup is shown in fig. S30.

### Interference analysis

To achieve controlled bending, the sensor’s two ends were attached to a stationary mount and the slider of a stepper motor (EB 1204 and CL-01A, HAIJIE Technology, Beijing). The bending degree was determined by the Arc-Chord Ratio (*L*_0_/*L*), comparing the final separation of the substrate ends after bending (*L*) to their original distance (*L*_0_). For pressure interference, the sensitive area of each hete-PE sensor was placed between two rectangular glass pads (1 cm by 0.8 cm) to ensure uniform load dispersion. A F305-IMT mechanical test system (MARK-10, Beijing) covering pressures of 0.5 to 500 N was then applied. Various humidity environments were established using saturated salt solutions at equilibrium—LiCl (11% RH), MgCl_2_ (33% RH), Mg(NO_3_)_2_ (54% RH), NaCl (75% RH), KCl (75% RH), and KNO_3_ (95% RH).

### Construction of biomimetic ionic skin

#### Preparation of gelatin-based hydrogel layer

To prepare the gelatin-based hydrogel solution, 2.0 g of gelatin particles was dissolved in 10.0 ml of DI water under continuous stirring at 60°C for 4 hours. Subsequently, 5.0 ml of glycerol was introduced, and the mixture was stirred at 60°C for an additional 2 hours to obtain the gelatin-based hydrogel precursor solution. The required amount of precursor was then cast onto a 10 cm–by–10 cm PTFE mold and allowed to cure at room temperature for 8 hours, forming a hydrogel film with ~20% surface moisture and a thickness of about 530 μm.

#### Preparation of biomimetic ionic skin

A flexible PI heater (1 cm by 1 cm), consisting of a serpentine metallic resistive pattern on the PI substrate, was attached to the back of the sensor (the side without the sensing material) so that it could maintain a human-body-like temperature. A customized gelatin-based hydrogel layer (1 cm by 1 cm) was then affixed to the sensing side as a “heat-dissipating dermal layer.” This assembly yielded a biomimetic thermal perception platform with a sandwich structure.

#### Fabrication of the hete-PE sensors array

To create the sensor array, flexible PI substrates featuring 5 by 5 electrode pixels were prepared. Each substrate (thickness ≈ 250 μm) carried electrodes composed of a 12-μm copper layer overlaid with 25-nm gold, with each pixel containing 11 pairs of interdigitated elements (width and spacing both 100 μm; length, 3 mm). Using the same drop-coating method, an appropriate volume of the hete-PE hydrogel precursor was applied to each electrode pixel. After the hydrogel had cured and was encapsulated with PTFE film, a 5 cm by 5 cm hete-PE sensor array was obtained. Last, this array was integrated with a flexible heater and a gelatin-based hydrogel layer to form a biomimetic thermal perception array in a sandwich configuration.

## Supplementary Materials

### This PDF file includes:

Text S1 to S7 Figs. S1 to S30 Tables S1 to S4 References
