# Supplementary material for: Energy landscape–engineered iontronics enable artificial thermoreceptors for augmented bioinspired thermosensation
Source: Sci Adv. 2025 Nov 7;11(45):eady2547. doi: 10.1126/sciadv.ady2547 (PMC12594173; doi:10.1126/sciadv.ady2547)
Supplement: Supplementary file 1 — Text S1 to S7 Figs. S1 to S30 Tables S1 to S4 References [file sciadv.ady2547_sm.pdf]

Science Advances
AAAS

Supplementary Materials for

Energy landscape–engineered iontronics enable artificial thermoreceptors for augmented bioinspired thermosensation

Fan Li *et al.*

Corresponding author: Hongran Zhao, zhaohr@jlu.edu.cn; Tong Zhang, zhangtong@jlu.edu.cn

Sci. Adv. **11**, eady2547 (2025)
DOI: 10.1126/sciadv.ady2547

This PDF file includes:

Text S1 to S7
Figs. S1 to S30
Tables S1 to S4
References

Text S1. Structural and Physical Characterization of hete-PE

(i) Materials

All chemicals were used as received without further purification unless otherwise noted. Vinyltrimethoxysilane (VTMS) and gelatin were obtained from Energy Chemical. Sodium vinylsulfonate (SVS, 25 wt% aqueous solution), glycerin (99%), acetone, and ammonia solution (28-30 wt%) were purchased from Aladdin Industrial Inc. Azodiisobutyrodinitrile (AIBN) was purchase from Sigma-Aldrich. N-methyl pyrrolidone (NMP) and poly(vinyl alcohol) (PVA-124) were sourced from Xilong Scientific Co., Ltd. Diethyl ether, methanol ethyl alcohol, ethyl acetate, and N,N-Dimethylformamide were acquired from Sinopharm Chemical Reagent Co., Ltd. Cellulose nanofibers (CNF, 1.5 wt% in water, diameter 5-20 nm, length 1-3 μm) were obtained from ScienceK Co., Ltd. Deionized water used throughout all experiments was purified via a millipore system. Edible liquid materials were purchased from Taobao (Alibaba Group, China).

(ii) Characterizations

Fourier transform infrared (FT-IR) spectra in the 400–4400 cm^{-1} range were acquired on a WQF-510A FTIR spectrometer (KBr as the matrix). The morphology and microstructure of samples were examined by field emission scanning electron microscopy (SEM, JSM-6700F) and transmission electron microscopy (TEM, JSM-2100F). Thermal gravimetric analysis (TGA) was performed on a PerkinElmer thermal analysis system up to 850 °C in air at a heating rate of 10 °C/min. The Young's modulus of polymer films was measured using a F305-IMT mechanical test system (MARK-10, Beijing) at a stretching rate of 5 mm/min (samples were cut to 5 cm × 1 cm).

(iii) Fabrication of self-healing hete-PE (hete-PE-SH) sensor

Type or paste text here. This should be additional explanatory text, such as: extended technical descriptions of results, full details of mathematical models, extended lists of acknowledgments, etc. It should not be additional discussion, analysis, interpretation, or critique.

(iv) Structural and Compositional Characterization of PEMS

To clarify the mesoscopic architecture of the hete-PE, we characterized the size, chemical composition, and elemental distribution of PEMS. Statistical analyses of SEM images (Fig. S2, A to C) revealed that the silica submicron sphere templates had a mean diameter of ~470 nm, while surface modification with PSSNa increased this to ~500 nm. TEM imaging combined with energy-dispersive X-ray spectroscopy (EDS) mapping (Fig. S2, D to G) confirmed a core–shell structure in PEMS, where sodium and sulfur from PSSNa were localized around the submicron sphere periphery. In the absence of an ion-transport medium, no spatial separation between sodium and sulfur was observed. FTIR spectroscopy (Fig. S3) further demonstrated successful surface modification: compared to pristine silica submicron spheres, PEMS displayed weakened peaks attributed to silanol (Si–OH) and C=C bonds, suggesting partial radical polymerization and the resultant grafting/encapsulation of PSSNa onto the submicron spheres.

(v) Quantification of PSSNa Content and Mechanical Properties

The average mass ratio of PSSNa to SiO_2 in PEMS was evaluated via TGA (Fig. S4). First, the residual mass fraction of Na_2SO_4 after heating pure PSSNa to 700 °C was ~54.60%, matching the theoretical combustion of PSSNa to Na_2SO_4 (54.57%). This served as a reference for PEMS decomposition (~26.00% weight loss at 700 °C), primarily due to the thermal decomposition of

PSSNa. From the relation $\Delta m/(m_{PSSNa}=45.40\%)$, the average PSSNa content in PEMS was calculated to be ~57.27 wt%. This allowed us to infer the actual PSSNa mass fraction in each hete-PE-1 to hete-PE-11 sample and to prepare corresponding homo-PE control groups (see Table S1).

The thermal stability and mechanical properties of hete-PE were further examined. TGA measurements indicated that hete-PE exhibited a decomposition onset temperature over 170 °C, ensuring its stability under standard operating conditions. The incorporation of PEMSs into the polymer matrix had a negligible effect on the Young's modulus of polymer matrix; however, an obvious increase in elongation at break (546%) was observed (Fig. S5). This enhancement aligns with previous studies showing that the inclusion of rigid fillers can improve the mechanical toughness of polymer networks (101).

Text S2. Interpretation of Charge Carriers in hete-PE

Ion transport in iontronic systems is inherently complex due to the diverse nature of ionic carriers, resulting in distinct charge transport mechanisms. In this study, both homo-PE and hete-PE are composite systems in which charge transport is dictated by their respective polymer matrices.

In the polyvinyl alcohol (PVA) matrix, a nonionic polymer, charge transport is primarily governed by proton hopping through adsorbed water molecules via the Grotthuss mechanism (equation (S1)). Upon the incorporation of PEMS, sodium ions (Na^+) dissociate from the sulfonate groups of PSSA, as described in equation (S2). These mobile sodium ions contribute to ionic conductivity through both diffusion and drift mechanisms.

$$2H_2O(\text{ads}) \rightleftharpoons H_3O^+ + OH^- \tag{S1}$$

$$RSO_3Na \rightleftharpoons RSO_3^- + Na^+ \tag{S2}$$

Since the contribution of proton conduction via the Grotthuss mechanism is negligible compared to sodium ion transport, we identify Na^+ as the dominant charge carrier in both homo-PE and hete-PE.

Text S3. Energy Landscape Model of Single Mesoscopic Structure in hete-PE

(i) Electrostatic Potential around a Fixed Anionic Core

Each fixed negative charged core (e.g., a negatively charged PSSNa submicron sphere) generates a spherically symmetric electrostatic potential $\psi(r)$. In equilibrium and in the absence of external fields, $\psi(r)$ satisfies the Poisson–Boltzmann equation, which relates the Laplacian of the potential to the local charge density. For a monovalent electrolyte and spherical symmetry, the nonlinear form can be written as:

$$\frac{1}{r^2}\frac{\mathrm{d}}{\mathrm{d}r}\left(r^2\frac{\mathrm{d}\psi(r)}{\mathrm{d}r}\right) = -\frac{q[c_+(r)-c_-(r)]}{\varepsilon_0\varepsilon_\mathrm{r}} = \frac{qc_0}{\varepsilon_0\varepsilon_\mathrm{r}}\left(\mathrm{e}^{\frac{-q\psi(r)}{k_\mathrm{B}T}} - \mathrm{e}^{\frac{+q\psi(r)}{k_\mathrm{B}T}}\right) \tag{S3}$$

where q is the cation charge (for cations), $c_+(r)/c_-(r)$ are the local concentrations of cations/anions, $\varepsilon_0\varepsilon_r$ is the permittivity of the medium, and k_BT is the thermal energy. In the weak potential regime ($|q\psi(r)| \ll k_BT$), we can linearize the equation to obtain the Debye–Hückel approximation:

$$\frac{1}{r^2}\frac{\mathrm{d}}{\mathrm{d}r}\left(r^2\frac{\mathrm{d}\psi(r)}{\mathrm{d}r}\right) \approx \kappa^2\psi(r) \tag{S4}$$

where κ is the Debye screening parameter, defined as $\kappa^2 = \frac{q^2 c_b}{\varepsilon_0 \varepsilon_r k_B T}$ for a monovalent cation cloud. Its inverse, $\lambda_D=1/\kappa$, is the Debye length, which sets the characteristic distance over which electrostatic interactions are screened. Solving the linearized equation in spherical coordinates yields the classic Debye–Hückel solution:

$$\psi(r) = \frac{Q_{core}}{4\pi\varepsilon_0\varepsilon_r} \frac{e^{-\kappa(r-r_0)}}{r}, r \geq r_0 \tag{S5}$$

where Q_{core} is the net charge of the anionic core (commonly negative) and r_0 is the core radius. This solution shows that $\psi(r)$ decays exponentially with r (multiplied by a $1/r$ geometric factor), reflecting the screening effect of surrounding counterions.

(ii) Boltzmann Distribution of Cations Around the Core

Mobile cations distribute themselves around a charged core following the Boltzmann distribution. In equilibrium without an external field, the local cation concentration at distance r from the core is

$$c(r) = c_0 \exp(-\frac{q\psi(r)}{k_B T}) \tag{S6}$$

where c_0 is a normalization constant (or far-field cation concentration). A more negative $\psi(r)$ boosts the exponential factor, causing higher cation concentration near the negatively charged core. If the core charge is large or the temperature is low, $\psi(r)$ is sufficiently deep and cations condense in the vicinity of the core; conversely, higher T or weaker core charge yields a flatter $\psi(r)$, leading to a more uniform distribution.

Substituting the Debye–Hückel potential solution shows that $c(r)$ assumes an exponential decay form. Physically, the ratio of electrostatic to thermal energy determines whether ions strongly condense near the core or remain more dispersed.

(iii) External Field and the Ion Escape Threshold Radius

For a cation to transport in hete-PE under an external electric field, that field must induce its escape from the electrostatic well. This involves surmounting an energy barrier, $E_{barrier}=U_{top}-U_{bottom}$. When the center-to-center distance d is large, their electrostatic wells remain isolated. However, when two submicron spheres are closer, their potential fields overlap: if the submicron sphere centers are near enough that their individual potentials superpose, the total potential does not reach zero at the nominal "edge" of each isolated well. Instead, the combined potential stays negative at what would have been the zero-potential boundary for a single sphere. Graphically, the "bottom" of each well is deeper, and the "rim" is lowered in energy by the neighbor's field contribution.

Consequently, the barrier $E_{barrier}$ that an ion must overcome to escape a single well is reduced compared to the energy barrier in a non-overlapping scenario. Because the "outside" region is already partially negative, the ion does not need to climb to zero potential to leave its original well. Quantitatively, many hopping models (e.g. correlated barrier hopping) show that reducing the distance between two Coulombic wells lowers the effective barrier from the isolated well depth to a smaller well depth. In our context, if the edge potential is no longer zero but instead $-\Delta\psi$, then the original barrier is reduced by $|q\Delta\psi|$. Hence, having nearby submicron spheres effectively "shallows" each potential well and creates a smoother conduction landscape.

When an external electric field E_{applied} is applied, each cation experiences a force qE_{applied}. This can liberate ions initially bound near the core. A cation escapes the well if the external field provides enough energy to overcome the net binding from the negative core. Formally, one solves

$$U(r) = q\psi(r) - qE_{\text{applied}}r \tag{S7}$$

finding a saddle point r_{min} where the inward Coulomb attraction balances the outward field force. Mathematically,

$$\left|\frac{\mathrm{d}\psi(r)}{\mathrm{d}r}\right|_{r=r_{\text{min}}} = E_{\text{applied}} \tag{S8}$$

Equivalently, in an energy-threshold form,

$$qE_{\text{applied}}\Delta x = E_{\text{barrier},i} \tag{S9}$$

where Δx is the relevant hop distance, and $E_{\text{barrier},i}$ is the energy difference from the cation's position at the saddle point to the top of the barrier.

For $r > r_{\text{min}}$, ($qE_{\text{applied}}\Delta x > E_{\text{barrier},i}$), the external field dominates, and ions can escape the core's potential well; for $r < r_{\text{min}}$,($qE_{\text{applied}}\Delta x < E_{\text{barrier},i}$), the core's binding remains stronger. Substituting the Debye–Hückel form of $\psi(r)$ gives an implicit equation for r_{min}.

(iv) Effective Ionic Charge Carriers of the Heterogeneous Polymer Electrolyte

Only cations that lie outside r_{min} and can respond to the external field contribute to long-range conduction. We define the effective charge-carrier concentration

$$c_{\text{eff}} = 4\pi\int_{r_{\text{min}}}^{r_{\text{top}}} c(r)r^2\mathrm{d}r \tag{S10}$$

where r_{top} approximates the outer boundary of the ion cloud. Higher temperature can increase c_{eff}, because thermal motion allows more ions to reach the region $r > r_{\text{min}}$.

(v) Effective Ion Mobility via the Einstein Relation

Freed ions (i.e., $r > r_{\text{min}}$) can migrate in the polymer electrolyte under the external field. Their mobility μ_{eff} is connected to the diffusion coefficient D by the Einstein relation:

$$\mu_{\text{eff}} = \frac{qD}{k_{\text{B}}T} \tag{S11}$$

Here, D is the ions' diffusion coefficient in the medium. Temperature raises μ_{eff} both by increasing D and by directly appearing in the denominator $k_{\text{B}}T$.

(vi) Ionic Conductivity of the Heterogeneous Polymer Electrolyte

The overall conductivity σ can be written as

$$\sigma = qc_{\text{eff}}\mu_{\text{eff}} \tag{S12}$$

analogous to the standard $\sigma = qc\mu$. Each factor has a clear physical role:

q: the cation's charge.

c_{eff}: the free-ion concentration, determined by electrostatic binding vs. field-driven release and thermal agitation.

μ_{eff}: the mobility of freed ions, tied to the diffusion coefficient D.

Overall, the model illuminates how temperature ($k_{\text{B}}T$), electrostatic binding (core charge, $\psi(r)$), and external field strength together govern ion transport. High temperature increases σ by enhancing both free-ion population (c_{eff}) and mobility (μ_{eff}). Conversely, strong binding (high core charge, low temperature) reduces σ by localizing ions near their cores. This framework, rooted in

Debye–Hückel theory and Boltzmann statistics, provides a quantitative picture of how a heterogeneous polyelectrolyte's microscale ion distribution translates into macroscale conductivity—qualitatively consistent with experimental observations in polymer electrolytes.

Text S4. Calculation of Thermal Index, Activation Energy (E_a), and Temperature Coefficient of Resistance (TCR)

The impedance modulus of the hete-PE exhibits strong temperature dependence, governed by a nonlinear relationship due to ion thermal activation mechanisms. This dependence follows an Arrhenius equation:

$$Z = Z_0 \exp(\frac{E_a}{k_B T}) \tag{S13}$$

where Z is the impedance modulus of hete-PE at temperature T, Z_0 represents the impedance modulus at an infinitely high temperature, and E_a is the thermal activation energy.

Taking the natural logarithm of equation S13 gives:

$$\ln(Z) = \ln(Z_0) + \frac{E_a}{k_B T} = \ln(Z_0) + \frac{B}{T} \tag{S14}$$

This linear relationship betweenln(Z) and $1/T$ allows for the determination of E_a from the slope of a ln(Z) vs. $1/T$ plot. The coefficient B known as the thermal index, is defined as:

$$B = \frac{E_a}{k_B} \tag{S15}$$

Furthermore, the TCR is given by:

$$\mathrm{TCR} = \frac{\mathrm{d}Z}{\mathrm{d}T} \times \frac{1}{Z} \tag{S16}$$

By combining equation S13 and S16, TCR can be expressed as:

$$\mathrm{TCR} = -\frac{B}{T^2} \tag{S17}$$

Text S5. Superposition of Electrostatic and Hydration-Induced Chemical Potentials in hete-PE

In addition to the nonuniform electrostatic potential generated by the negatively charged cores, the difference in hydrophilicity of the PVA matrix and PSSNa domains gives rise to spatial variations in water distribution, which also contribute to the ionic energy states through hydration-induced chemical potentials. For the sake of modeling simplicity, these chemical potential effects are treated as a background superimposed on the electrostatic potential. Because the hydroxyl groups in the PVA matrix are uniformly distributed and exhibit only weak polarization charges without strong binding to sodium ions, PVA primarily serves as a uniform dielectric–viscoelastic background and does not perturb the energy landscape governed by the negatively charged cores. In contrast, the stronger hydrophilicity of PSSNa leads to local water enrichment in the vicinity of polyelectrolyte submicron sphere, whereas the homo-PE exhibits a nearly homogeneous hydration profile. Since regions of higher water content correspond to lower ionic chemical potential, the influence of heterogeneous hydration on the ionic energy landscape is consistent with the radial electrostatic potential gradient generated by the negatively charged cores. Because the PSSNa

content in hete-PE is low (<4 wt%), the impact of hydration heterogeneity on the energy landscape is negligible compared with the electrostatic contribution and is not discussed further. As its spatial variation parallels the radial electrostatic gradient, this effect is implicitly incorporated into the overall transport model.

Text S6. Interpretation of Nyquist plot and Bode plot

Fig. S9-S11 present the Nyquist and Bode plots of the system. Given the absence of redox reactions at the electrode-electrolyte interface, charge transfer resistance between the electrode and electrolyte can be disregarded. The equivalent circuit consists of a constant phase element (*CPE*) associated with the electrical double layer (CPE_{EDL}), bulk resistance (R_B), bulk capacitance (geometrical capacitance, (C_B), and electrode resistance (R_E).

Due to the pronounced mass transport effects of ionic carriers, CPE_{EDL} and R_B exhibit distinct relaxation phenomena when the AC frequency (f) surpasses their characteristic relaxation frequencies. Consequently, the equivalent circuit can be simplified into different configurations across frequency regimes, revealing that ion transport dynamics in the polymer electrolyte are governed by distinct diffusion, migration, and polarization processes.

(i) Nyquist Plot Analysis

Type or paste text here. This should be additional explanatory text, such as: extended technical descriptions of results, full details of mathematical models, extended lists of acknowledgments, etc. It should not be additional discussion, analysis, interpretation, or critique.

-Low-Frequency Region ($f < f_D$)

The inflection point at the transition from the semicircle to the linear region corresponds to the characteristic dielectric relaxation frequency (f_D), which is associated with interfacial charge polarization. At frequencies below f_D, the electrical double layer (EDL) forms at the electrode interface. Since the EDL capacitance (C_{EDL}) is much larger than C_B, and R_B is much smaller than the reactance of C_B ($1/(2\pi f C_B$)), the overall impedance behavior is dominated by the C_{EDL}, with contributions from R_B and R_E. This indicates that, at low frequencies, the electrical behavior of the polymer electrolyte is primarily governed by interfacial polarization processes.

-Mid-Frequency Region ($f_D < f < f_C$)

In this regime, ions can no longer accumulate at the electrode interface to form the stable EDL, leading to a transition in the equivalent circuit, which simplifies to a parallel combination of R_B and C_B, in series with R_E. Since R_B remains smaller than $1/(2\pi f C_B$), charge transport in the polymer electrolyte is primarily dictated by ion migration.

-High-Frequency Region ($f > f_C$)

Above the characteristic charge relaxation frequency (f_C), ion migration can no longer keep pace with the alternating electric field, leading to pronounced charge relaxation effects. Consequently, the electrical behavior of the polymer electrolyte becomes dominated by the dielectric properties of the PVA matrix rather than ion transport.

(ii) Bode Plot Analysis

The distinct ion transport mechanisms observed in the Nyquist plot are further reflected in the Bode plots.

-Impedance Modulus (|*Z*|) Bode Plot
In both the high-frequency and low-frequency regions, the logarithm of the impedance modulus exhibits a linear relationship with the logarithm of frequency, characteristic of capacitive behavior ($\log|Z|=\log(1/2\pi C)-\log f$) (Fig. S11A). In contrast, in the mid-frequency region, the curve remains nearly parallel to the x-axis, indicating resistive behavior.

-Phase Angle Bode Plot
The phase angle transitions from -90° in the low-frequency regime (dominated by interfacial polarization) to 0° in the mid-frequency regime (where ion migration dominates) and then back to -90° in the high-frequency regime (where bulk dielectric relaxation prevails) (Fig. S11B). This behavior is consistent with the transition from interfacial polarization at low frequencies to ion transport at intermediate frequencies, followed by dielectric relaxation at high frequencies.

-Capacitance Bode Plot
Above f_D, a distinct dielectric loss phenomenon emerges, further corroborating the presence of interfacial polarization relaxation (Fig. S11C). This behavior aligns with the dielectric relaxation associated with interfacial polarization.

Text S7. Simulation Methods and Parameters

Numerical simulations were performed using MATLAB to provide supporting evidence for Fig. 1A, Fig. 1E, and Fig. 2B. The simulations were based on simplified Debye–Hückel (DH) model. For a single negatively charged spherical domain, the electrostatic potential was calculated based on equation S5. The cation concentration was obtained using the Boltzmann distribution (equation S6). For two-domain systems, the potential distribution was computed by superimposing two DH potentials (ψ_{DH}) from two spheres separated by distance *d*:

$$\psi(x,y)=\psi_{\mathrm{DH}}(|r-r_1|)+\psi_{\mathrm{DH}}(|r-r_2|) \tag{S18}$$

where r_1 and r_2 are the distances to each sphere center. The cation potential energy was then obtained as $E = qe\psi$, expressed in units of k_BT.
Parameters used unless otherwise noted:
Sphere radius: r_0 = 250 nm
Fixed core charge: Q_{core} = - 200*e*
Relative permittivity: ε_r = 40
Far-field cation concentration: c_0 = 0.11 mM
Grid resolution: 1200 radial points (single-sphere); 501×401 mesh points (two-sphere maps)
Inter-sphere spacings: 5000, 1500, 900 nm
Limitations: Several parameters (e.g., core charge, dielectric constant, and background ion concentration) are difficult to determine precisely because the system is intrinsically heterogeneous and the surface groups on polyelectrolyte microspheres are only partially ionized. Therefore, the parameters were assigned within reasonable ranges. The simulations are not quantitatively predictive but capture the qualitative trends of ionic redistribution, thermal broadening, and potential well overlap, providing valuable support for the schematics.

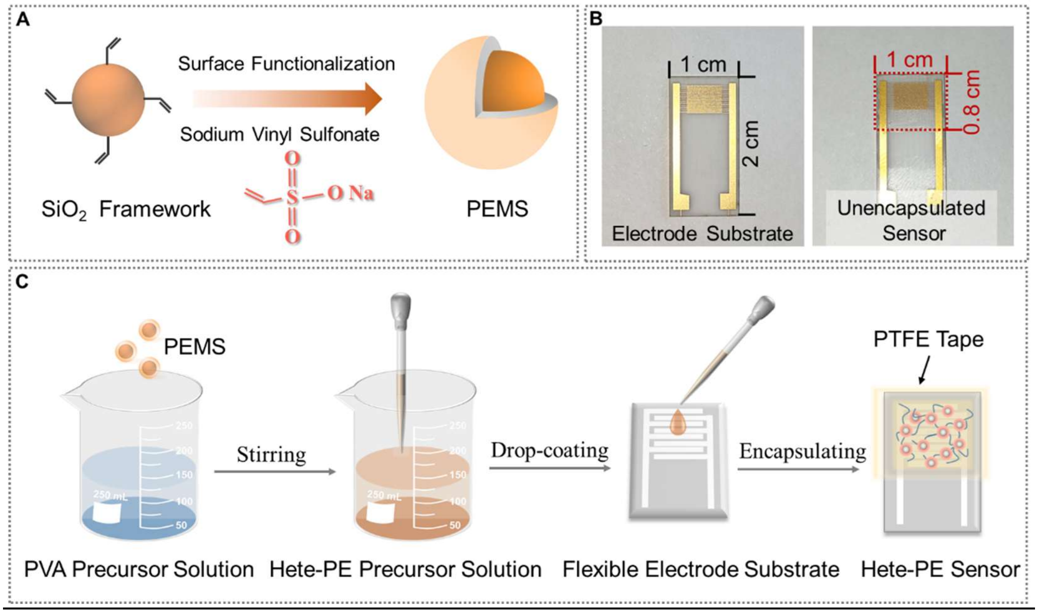

Fig. S1. Synthesis of hete-PE and sensor fabrication.

(A) Schematic of the synthesis process of PEMSs. **(B)** Optical photos of the flexible electrode substrate and an unencapsulated hete-PE sensor; the active layer spans ~0.8 cm^2 with a thinkness of ~130 μm. **(C)** Schematic illustration of the preparation of hete-PE sensor.

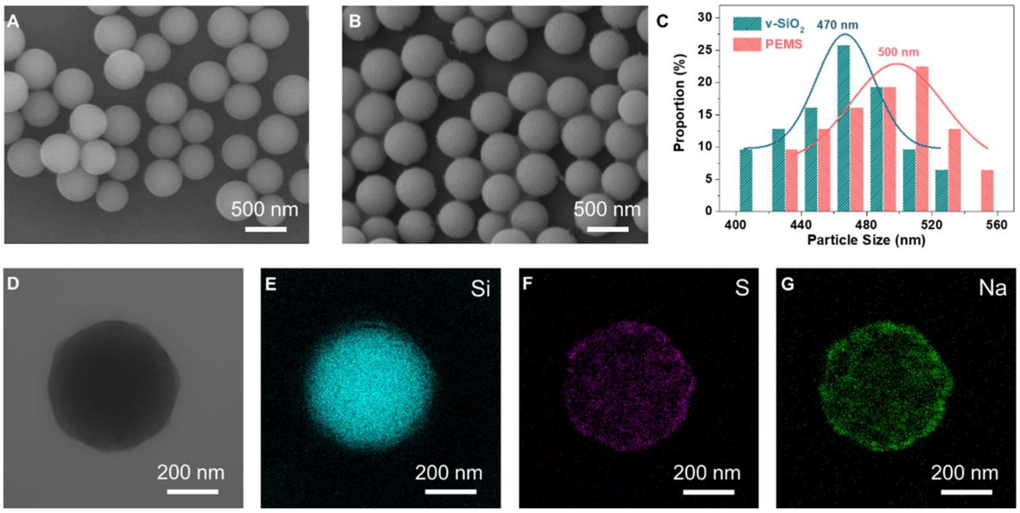

Fig. S2. Characterization of the structure of PEMS.

(A and B) SEM images of vinyl functionalized SiO_2 (v-SiO_2) (A) and PEMS (B). **(C)** Particle size distributions for v-SiO_2 and PEMS, indicating a diameter shift from ~470 nm to ~500 nm upon surface functionalization. **(D-G)** TEM image of a single PEMS particle (D) and corresponding EDS mapping of silicon (Si), sulfur (S), and sodium (Na).

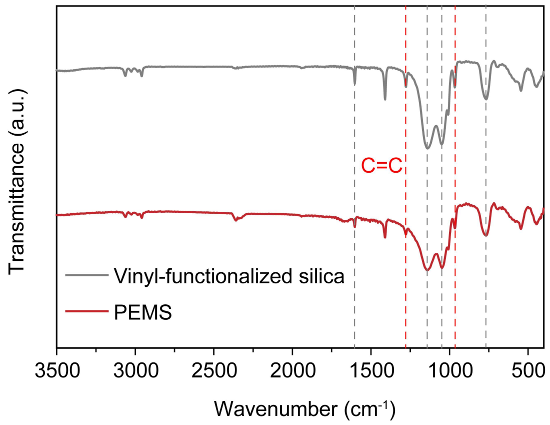

Fig. S3. FT-IR spectra of vinyl-functionalized silica and PEMS.

The peak at 1602 cm^{-1} is attributed to Si–OH stretching, while the peaks at 1135 cm^{-1} and 1046 cm^{-1} indicate asymmetric Si–O–Si stretching. Notably, the peak near 1280 cm^{-1} and 966 cm^{-1}, associated with C=C bonds, appear in both spectra, though their intensity in PEMS is weakened due to partial consumption of C=C during grafting.

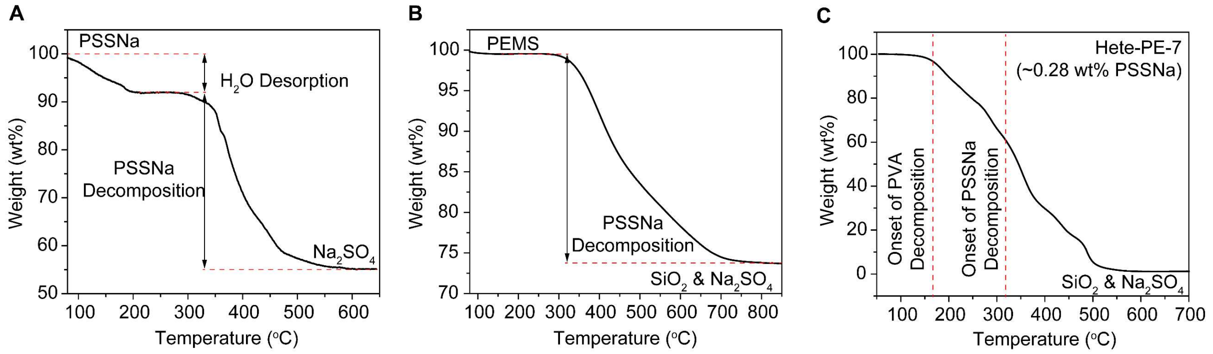

Fig. S4. Thermogravimetric analysis of PSSNa, PEMS, and hete-PE-7.

(A and B) Thermogravimetric analysis (TGA) of PSSNa (A), and PEMS (B), both heated at 10 °C/min from 80 °C to 850 °C. For PSSNa, the mass loss below 200 °C corresponds to water evaporation, while the main thermal decomposition near 350 °C leaves Na_2SO_4. In PEMS, the final residue consists of SiO_2 and Na_2SO_4. **(C)** TGA of hete-PE-7 heated at 10 °C/min from 50 °C to 700 °C. The drop around 150 °C reflects PVA matrix decomposition, followed by an accelerated mass loss near 350 °C as the PEMS component decomposes. Above 500 °C, pyrolysis completes, leaving SiO_2 and Na_2SO_4.

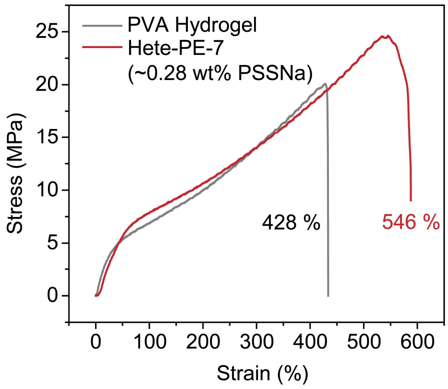

Fig. S5. Stress-strain curves of PVA hydrogel and hete-PE-7.

Both materials exhibit similar Young's modulus (~5.5 MPa), while their tensile strengths are ~20.1 MPa for the PVA hydrogel and ~24.6 MPa for hete-PE-7. In addition, hete-PE-7 shows a larger elongation at break (> 540%) compared with the PVA hydrogel (~420%).

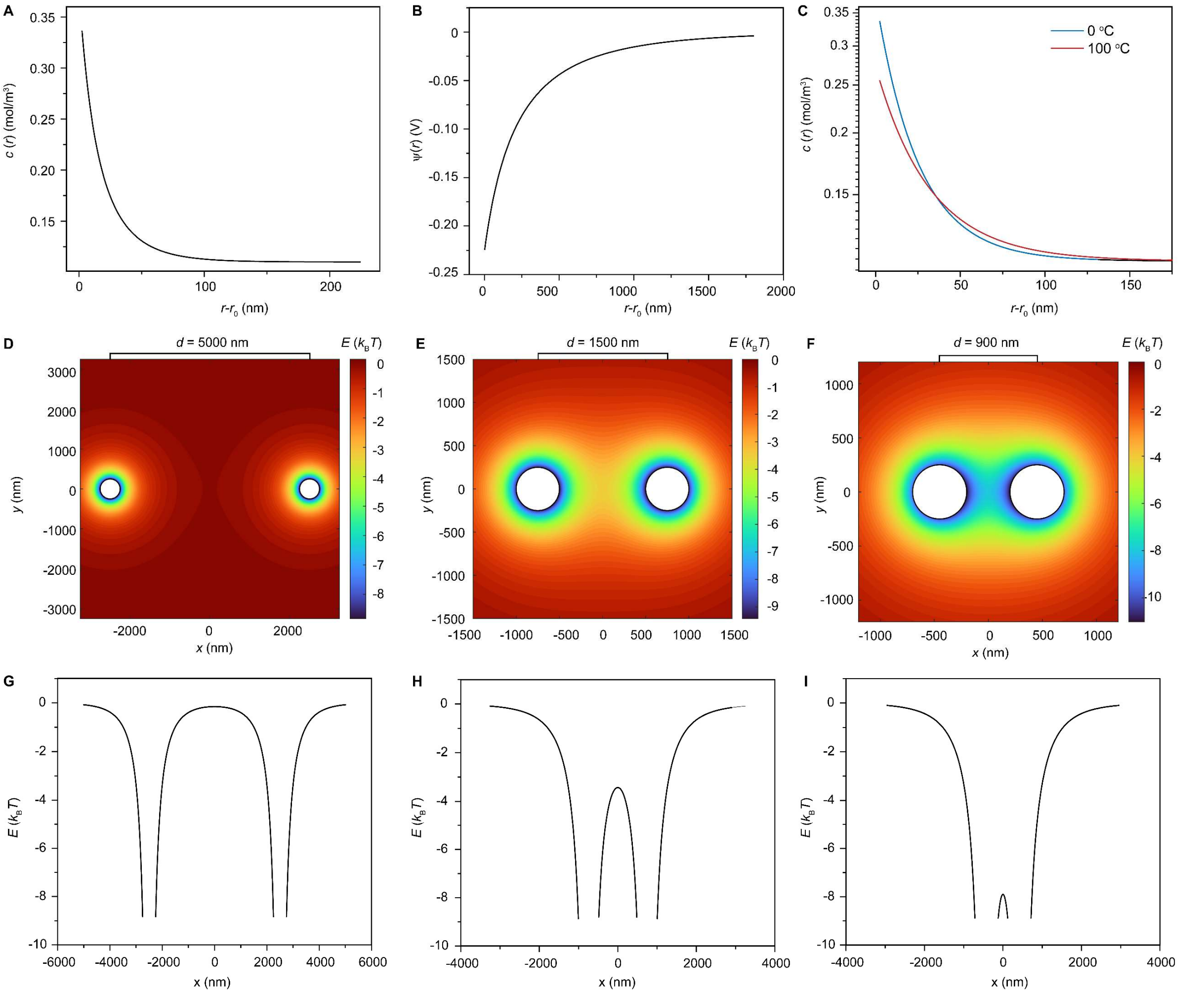

Fig. S6. Simplified Debye–Hückel simulations of hete-PE.

(A) Radial cation concentration distribution around a single negatively charged core. **(B)** Corresponding radial potential profile. **(C)** Comparison of cation concentration profiles at 0 °C and 100 °C. **(D–F)** Two-sphere cation potential energy maps at center-to-center spacings of 5000 nm (D), 1500 nm (E), and 900 nm (F). **(G–I)** Potential energy profiles along the inter-sphere axis for center-to-center spacings of 5000 nm (G), 1500 nm (H), and 900 nm (I).

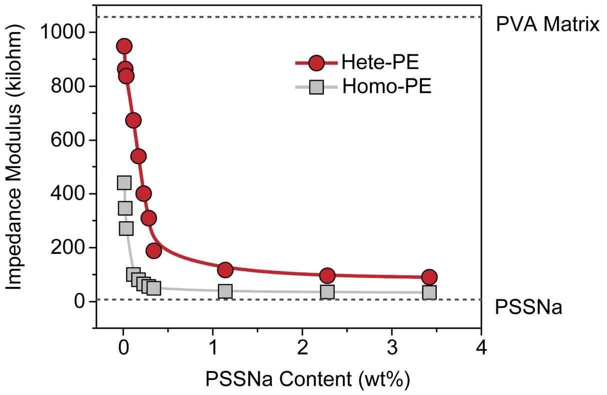

Fig. S7. Impedance modulus of hete-PE and homo-PE with varying PSSNa content.

Both structurally heterogeneous (hete-PE) and homogeneous (homo-PE) polymer electrolytes exhibit impedance variations with PSSNa concentration that are in agreement with Kohlrausch's law of ionic conduction.

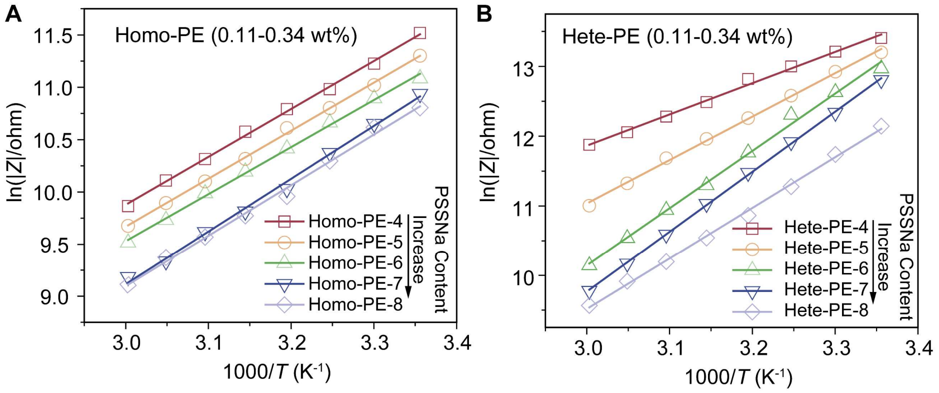

Fig. S8. Arrhenius analysis of ionic conduction in homo-PE and hete-PE.

(A) Arrhenius plot of ln|Z| versus 1000/T for homo-PE with PSSNa contents of 0.11–0.34 wt%.
(B) Corresponding Arrhenius plot for hete-PE over the same PSSNa concentration range.

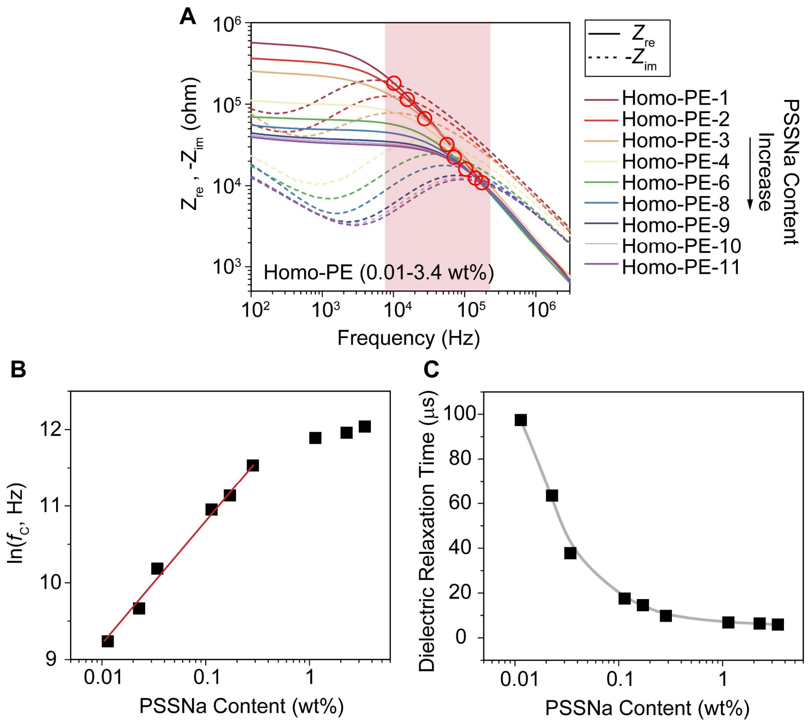

Fig. S9. Impedance spectra and charge relaxation behavior of homo-PE with varying PSSNa content (0.01–3.4 wt%).

(A) Real (Z_{re}, solid line) and imaginary (Z_{im}, dotted line) components of the impedance as a function of frequency. **(B and C)** charge relaxation frequency (B) and dielectric relaxation time (C) of homo-PE with different PSSNa contents.

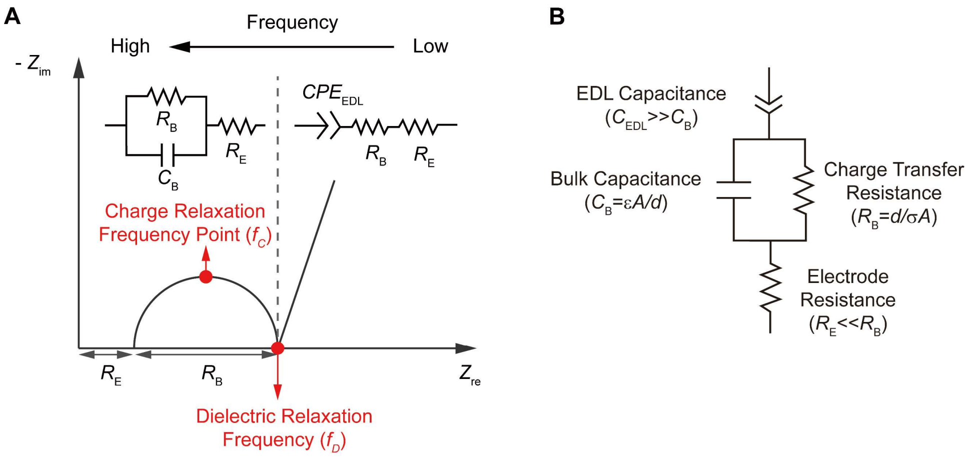

Fig. S10. Conceptual illustration of complex impedance spectroscopy (CIS) and its equivalent circuit.

(A) Schematic Nyquist plot highlighting the and the three distinct regions divided by charge relaxation frequency (f_C) and the dielectric relaxation frequency (f_D), corresponding dominant circuit components are denoted in the Nyquist plot. **(B)** Simplified equivalent circuit capturing electrode resistance (R_E), bulk resistance (R_B), bulk capacitance (C_B), and electrical double-layer (EDL) capacitance (C_{EDL}).

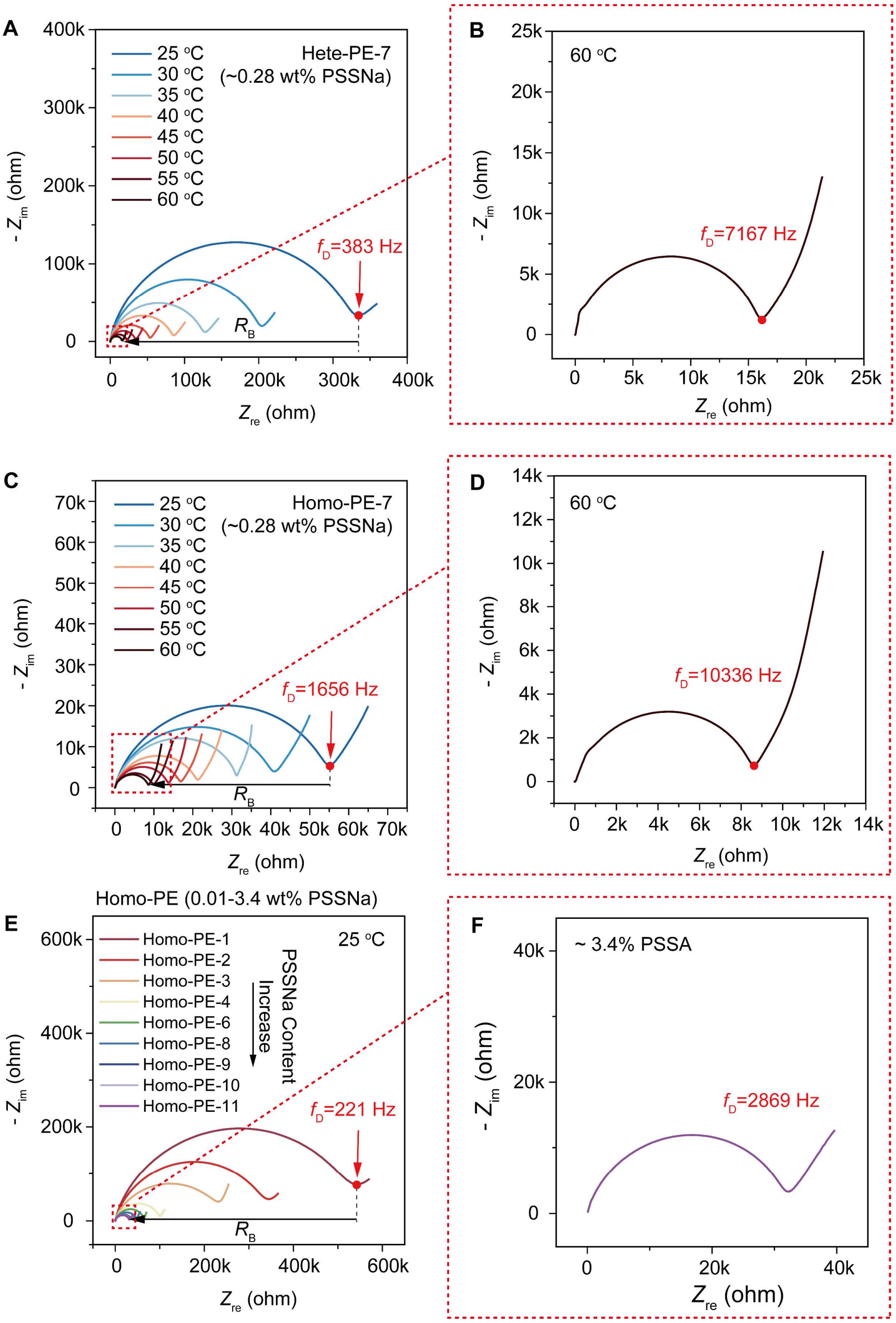

Fig. S11. Nyquist plots of homo-PE and hete-PE.

(A–D) Nyquist plots of hete-PE-7 and homo-PE-7 measured between 25 and 60 °C. **(E and F)** Nyquist plots of homo-PE with PSSNa contents ranging from 0.01 to 3.4 wt%.

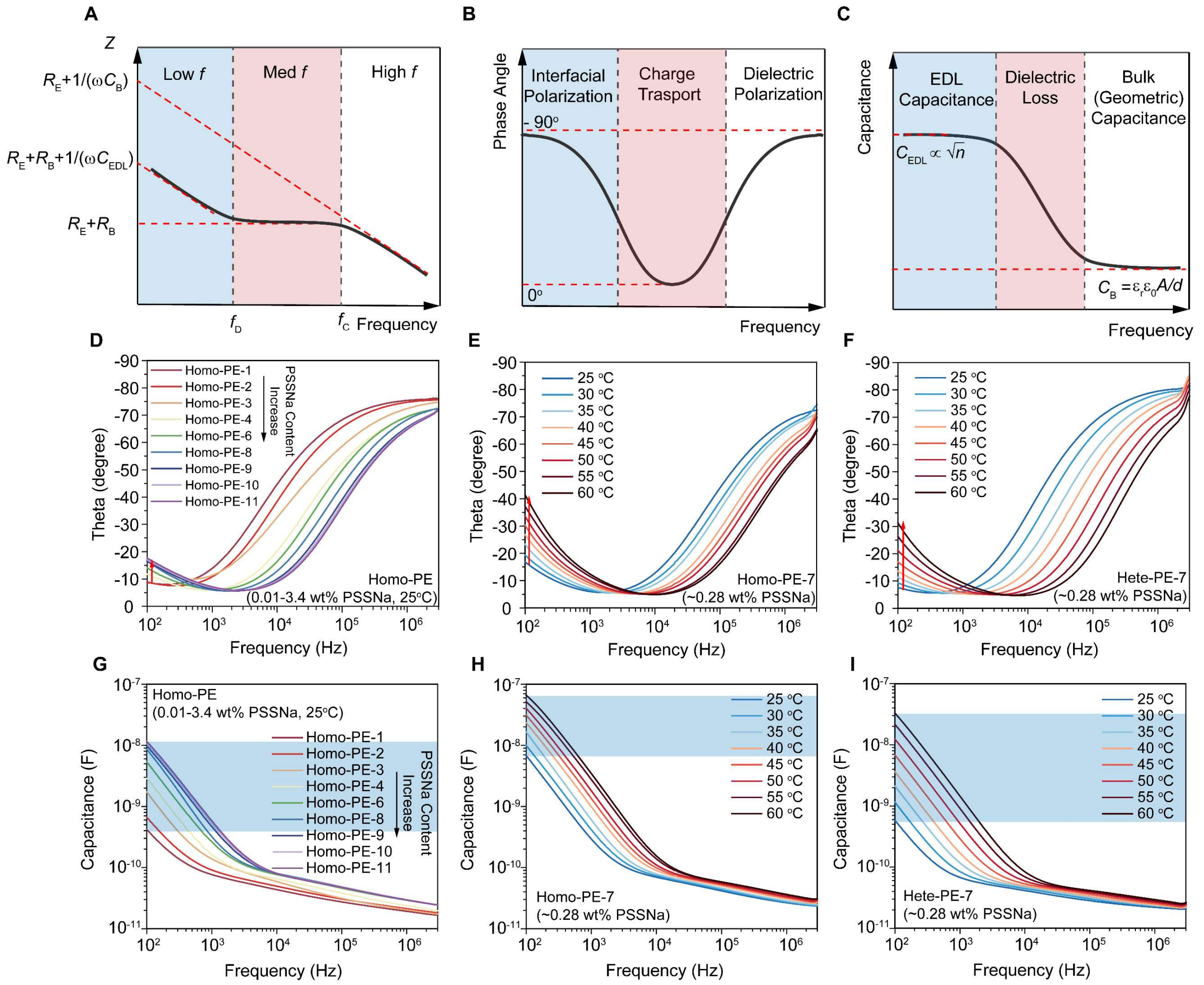

Fig. S12. Bode Plots of hete-PE and homo-PE.

(A–C) Schematic Bode plots depicting ionic behavior across frequency ranges. Here, f_D is the dielectric polarization frequency and f_C is the charge relaxation frequency. **(D)** Frequency-dependent phase angles of homo-PE with different PSSNa content. **(E and F)** Frequency-dependent phase angles of (E) homo-PE-7 and (F) hete-PE-7 at different temperature (25–60 °C). **(G)** Frequency-dependent capacitance curves for homo-PE with different PSSNa loadings. **(H and I)** Capacitance versus frequency for (H) homo-PE-7 and (I) hete-PE-7 at different temperature (25–60 °C). The more pronounced temperature-driven ionic concentration changes in hete-PE-7 lead to notably larger capacitance variations compared with homo-PE-7.

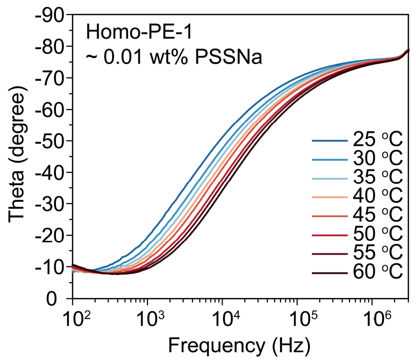

Fig. S13. Frequency-dependent phase angles of homo-PE-1 (0.01 wt% PSSNa) at different temperatures.

Phase angle spectra were measured over 25–60 °C. In the low-frequency region, the phase angles of homo-PE-1 show negligible dependence on temperature, indicating stable interfacial polarization behavior.

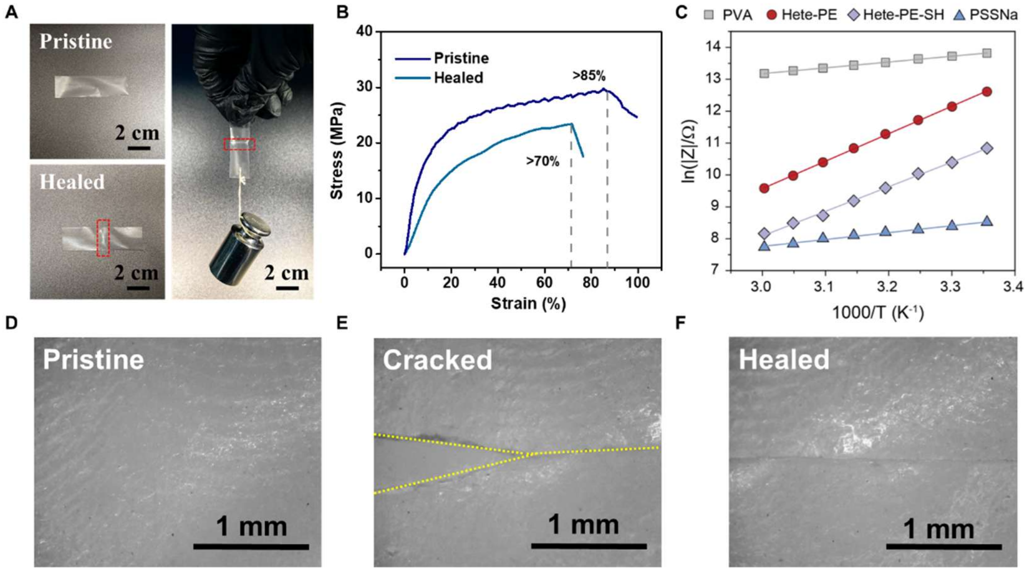

Fig. S14. Self-healing performance, mechanical properties, and temperature-sensing characteristics of hete-PE-SH.

(A) Optical photos of pristine (top) and self-healed (bottom) samples. At right, a self-healed film easily supports a 100 g weight. **(B)** Stress–strain curves before and after healing, showing ~82% retention of tensile strength. **(C)** Arrhenius plots for PVA, hete-PE, hete-PE-SH, and PSSNa. hete-PE-SH exhibits higher conductivity than hete-PE and retains nearly parallel slopes, indicating minimal compromise in temperature sensitivity. **(D-F)** Optical microscope photos of a sample in pristine (D), cracked (E), and healed states (F), respectively.

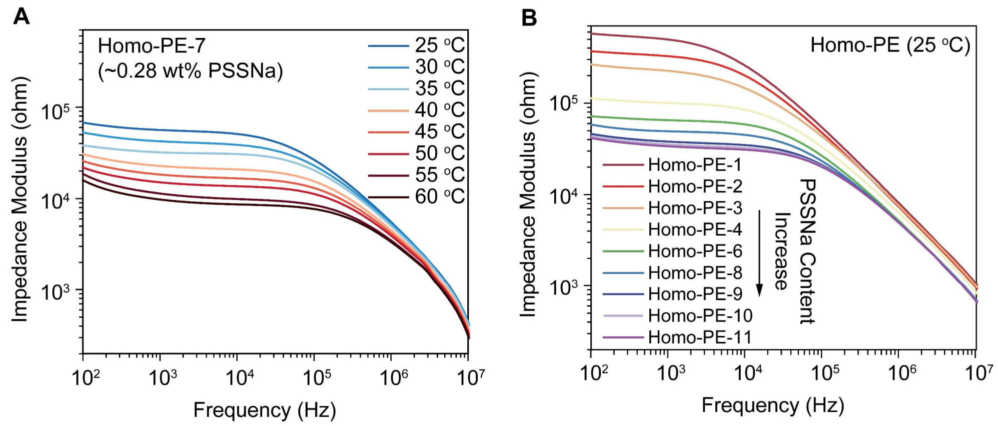

Fig. S15. Frequency dependence of impedance modulus for homo-PE.

(A) Curves acquired at different temperatures (25–60 °C) for homo-PE-7 (~0.28 wt% PSSNa). **(B)** Curves acquired at 25 °C for homo-PE samples with varying PSSNa contents.

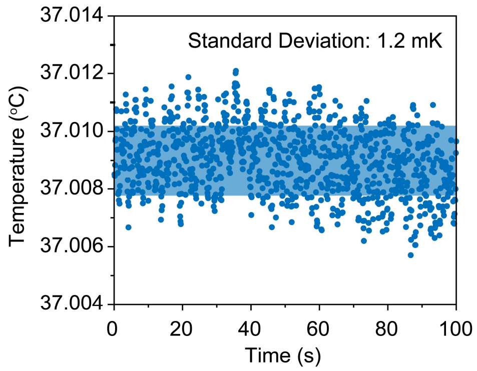

Fig. S16. Baseline noise analysis of the hete-PE-7 sensor at 10 Hz.

Measured over 100 s in a thermally insulated enclosure, the sensor's output shows a standard deviation of ~1.2 mK, representing its theoretical sensitivity limit.

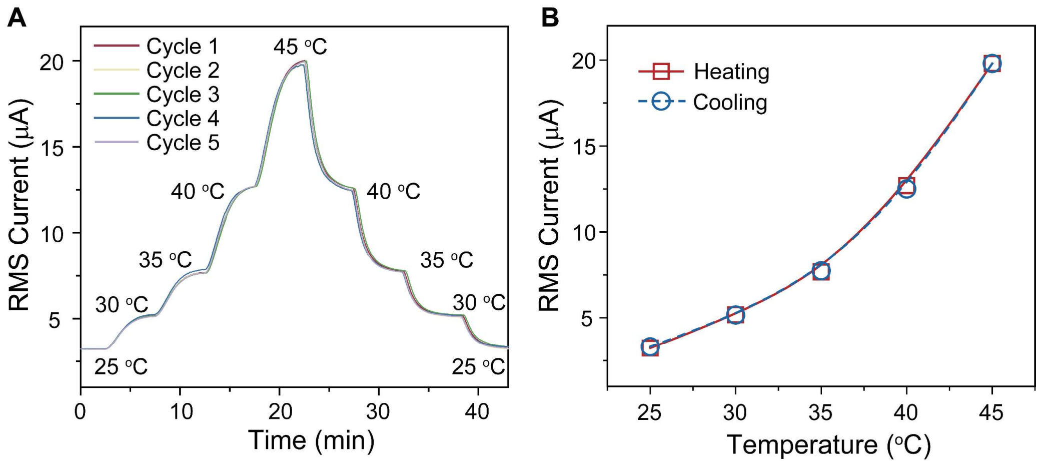

Fig. S17. Temperature hysteresis of the hete-PE-7 sensor.

(A) Continuous five-cycle heating–cooling response of the hete-PE-7 sensor over 25–45 °C with a 5 °C step. **(B)** Temperature hysteresis plot obtained by extracting the mean equilibrium current values from the five heating–cooling cycles at each step, showing negligible difference between the heating (red squares) and cooling (blue circles) processes.

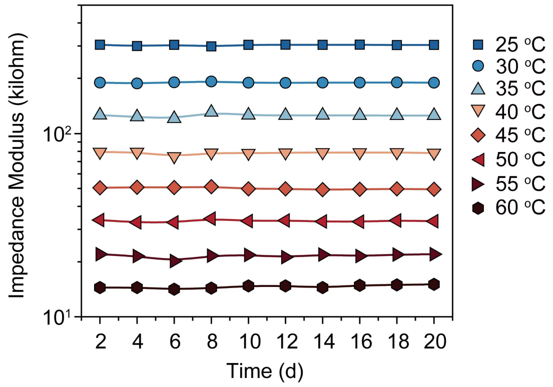

Fig. S18. Long-term stability of the hete-PE-7 sensor.

Plotted is the impedance modulus measured at temperatures from 25 to 60 °C over 20 days, demonstrating minimal drift.

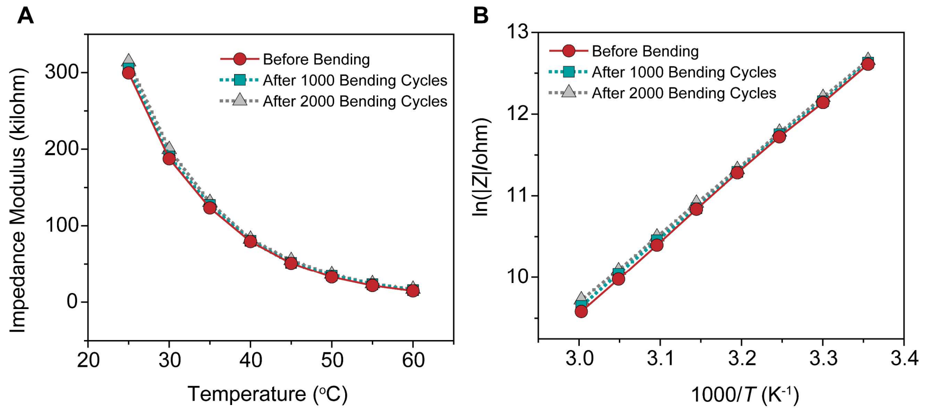

Fig. S19. Mechanical robustness of hete-PE-7.

(A) Impedance modulus–temperature curves before bending and after 1000 and 2000 bending cycles. **(B)** Corresponding Arrhenius plots, showing minimal deviation even after repeated deformations.

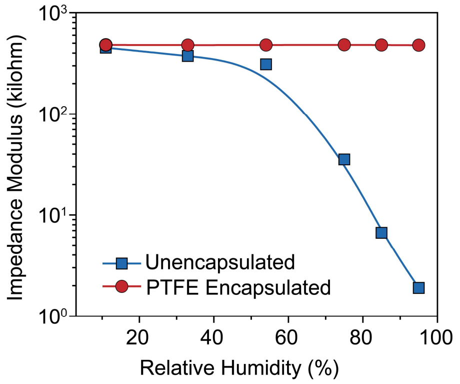

Fig. S20. Humidity-dependent impedance modulus of hete-PE-7.

Impedance modulus was measured at 25 °C across 11–95% RH. PTFE encapsulation effectively suppresses humidity-induced variations compared with the unencapsulated sensor.

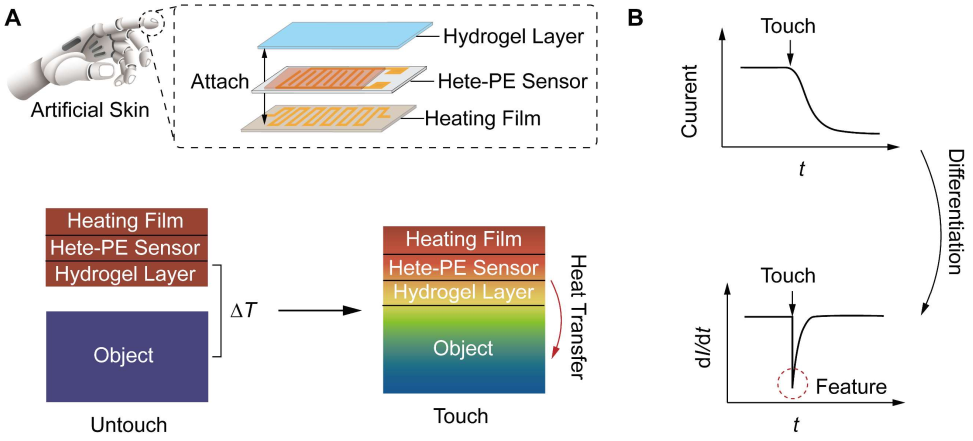

Fig. S21. Schematic of the biomimetic thermal-tactile approach for solid identification.

(A) Exploded view of the ionic skin architecture—comprising a heating film, hete-PE sensor, and hydrogel layer—and the heat transfer process upon contact with an object at lower temperature. **(B)** Representative current–time (top) and differentiated current–time (bottom) curves when the ionic skin contacts a room-temperature object. The peak in the differentiated signal serves as the feature value for material recognition.

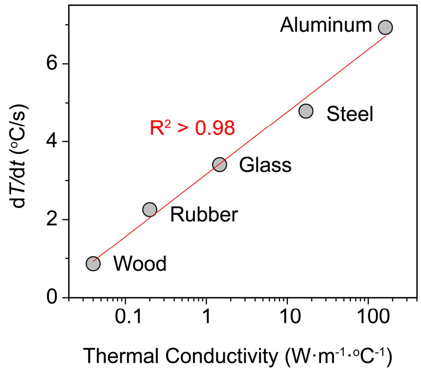

Fig. S22. Ionic skin response as a function of material thermal conductivity.

Correlation between the ionic skin's feature value and the thermal conductivity of various materials.

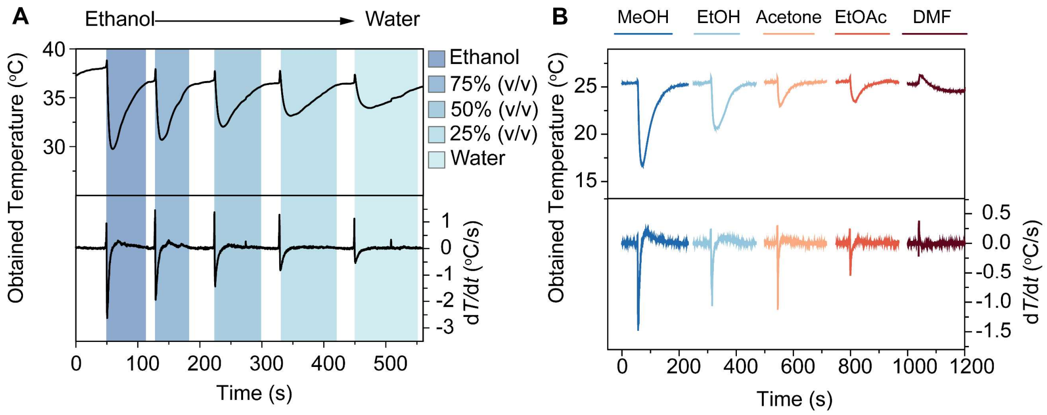

Fig. S23. Temperature response of ionic skin and hete-PE-7 to liquids of different compositions.

(A) Time - resolved temperature curves (top) and their first derivatives (bottom) measured by the biomimetic ionic skin when contacting ethanol – water mixtures of various volume ratios. **(B)** Corresponding data for the Hete - PE - 7 sensor touching different solvents.

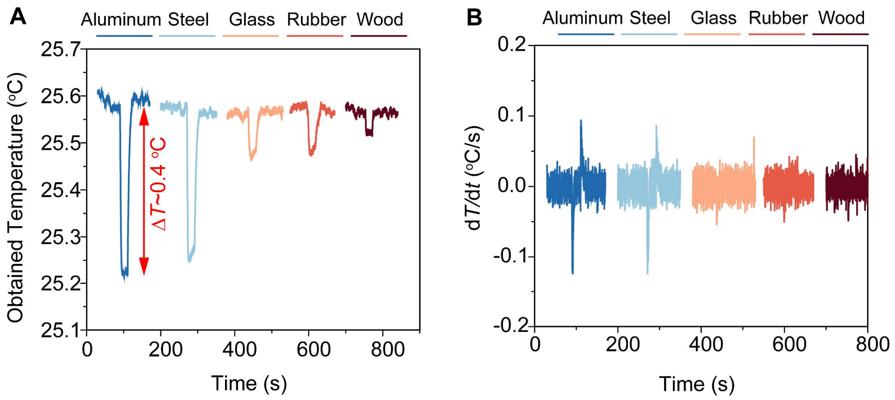

Fig. S24. Thermal response of the hete-PE-7 sensor in contact with solids of different thermal conductivities.

(A) Time - resolved temperature responses of the hete - PE - 7 sensor when touching aluminum, steel, glass, rubber, and wood blocks, each at the same ambient temperature (~25 °C). **(B)** Corresponding first - derivative curves of hete-PE-7 sensor touching with different cubes.

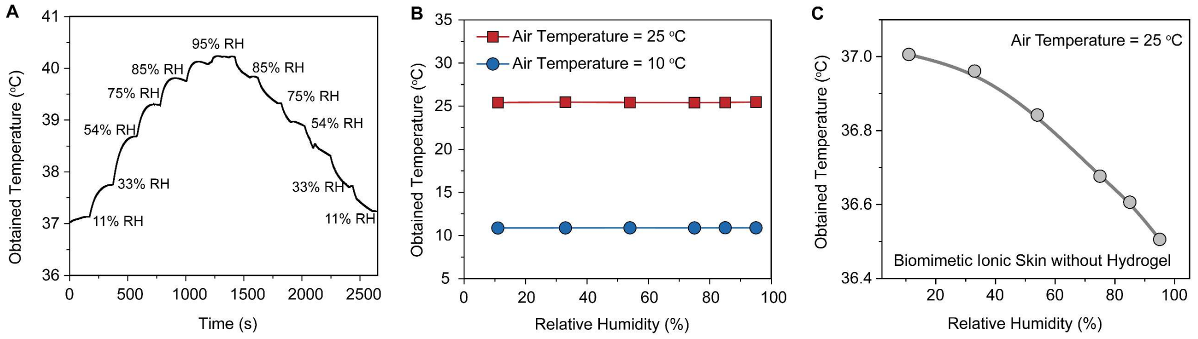

Fig. S25. Humidity-dependent obtained temperature response of the ionic skin and hete-PE-7 sensor.

(A) Time evolution of the ionic skin's obtained temperature as relative humidity cycles from 11 % to 95 % RH at 25 °C. **(B)** Variation in obtained temperature of hete-PE-7 with increasing humidity at 10 °C and 25 °C, respectively. **(C)** Obtained temperature of biomimetic ionic skin without hydrogel as the relative humidity increases from 11 to 95% RH.

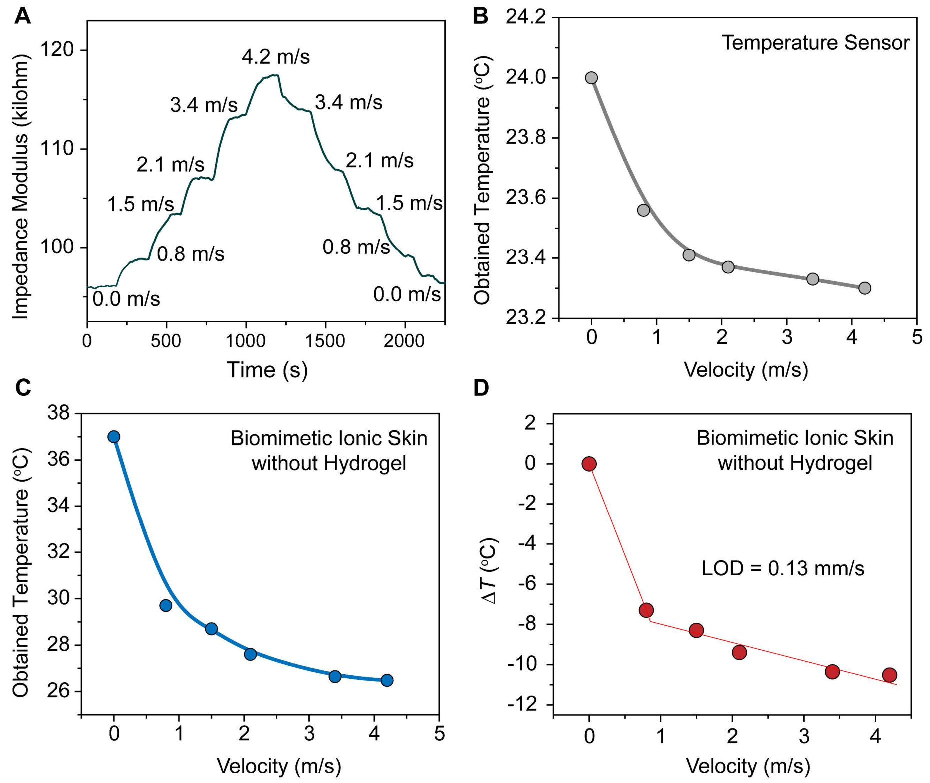

Fig. S26. Airflow-dependent obtained temperature of the ionic skin and hete-PE-7 sensor.

(A) Dynamic variation of the ionic skin's impedance modulus under airflow speeds ranging from 0.0 to 4.2 m/s at 25 °C. **(B)** Obtained temperature of a stand-alone hete-PE-7–based thermoreceptor across different airflow velocities. **(C)** Temperature profile of the ionic skin without a hydrogel layer as airflow velocity increases. **(D)** Corresponding net temperature change of the hydrogel-free ionic skin, exhibiting a theoretical detection limit of 0.13 mm/s.

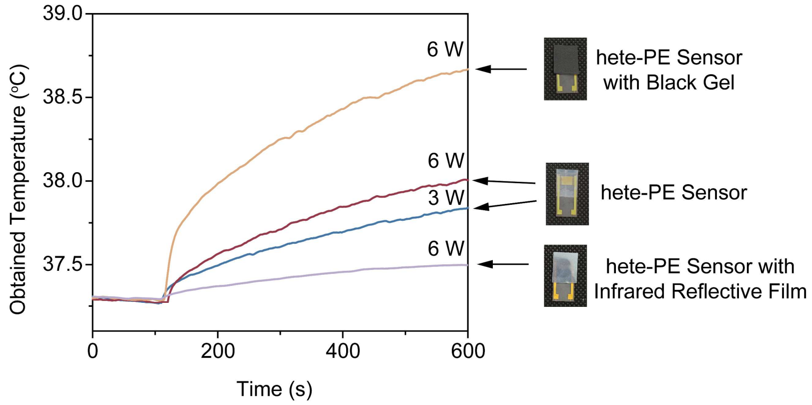

Fig. S27. The solar heating response of a hete-PE-7 sensor.

Real-time temperature evolution of a hete-PE-7 sensor under sunlamp illumination, comparing different surface coatings. The sensor coated with a black gel exhibits greater infrared absorption and thus a larger temperature rise, whereas the one covered by an infrared-reflective film shows minimal heating due to reduced IR penetration and convective cooling.

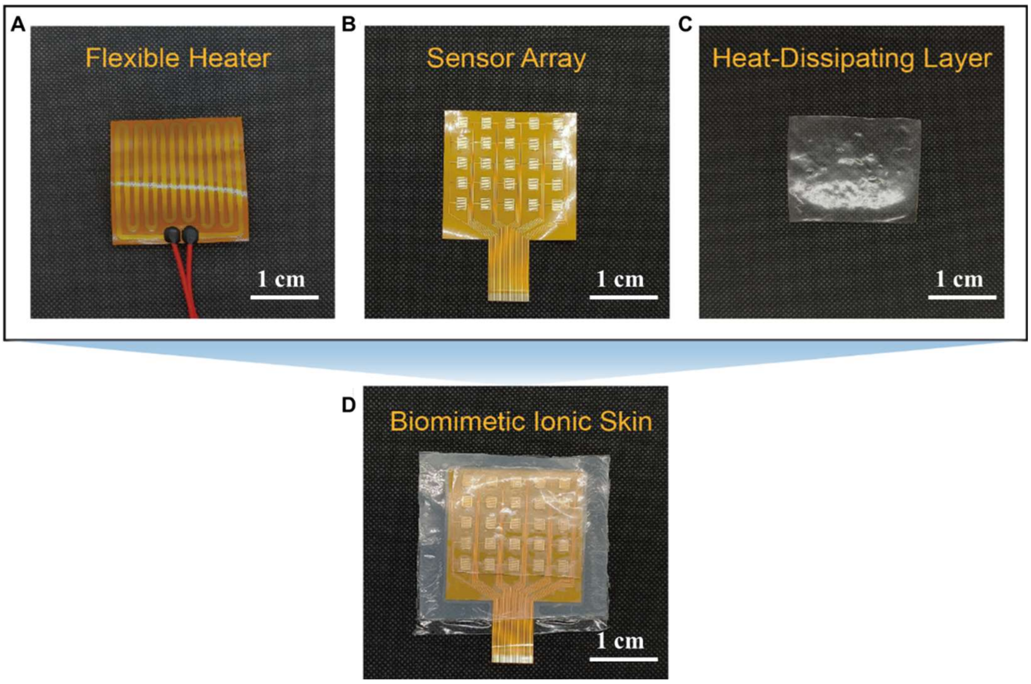

Fig. S28. Structural components of the biomimetic ionic skin.

(A–D) Photographs of the flexible heater (A), the sensor array (B), the heat-dissipating layer (C), and the assembled biomimetic ionic skin (D). The device adopts a sandwich structure, with the heat-dissipating layer on top, the sensor array in the middle, and the flexible heater at the base.

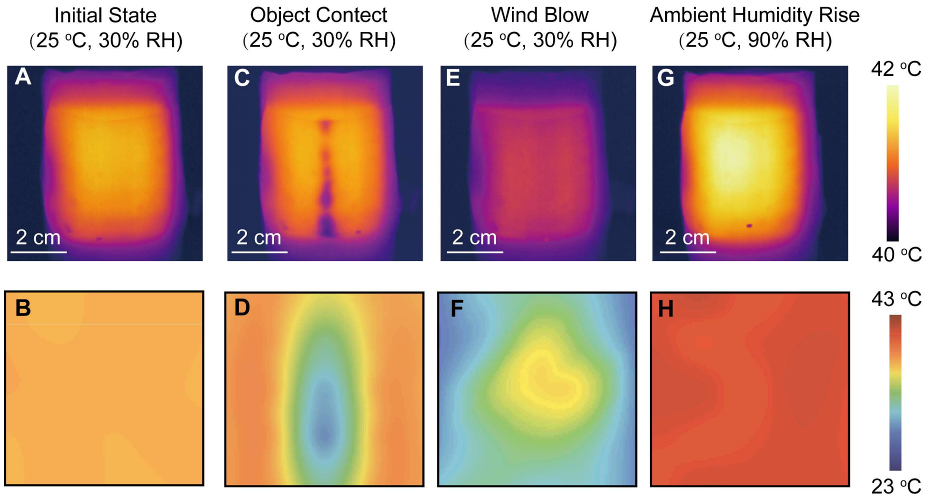

Fig. S29. Infrared thermograms and 2D temperature maps of the biomimetic ionic skin under different environmental stimuli.

Infrared thermograms **(A, C, E, G)** and corresponding 2D temperature maps **(B, D, F, H)** of the biomimetic ionic skin, generated via the random (Renka–Cline) interpolation method, in four distinct scenarios: initial state (25 °C, 30% RH), contact with a room-temperature object (25 °C, 30% RH), exposure to unidirectional airflow (25 °C, 30% RH), and an elevated ambient humidity (25 °C, 90% RH). The 2D maps provide a detailed spatial view of temperature variations, highlighting how the ionic skin responds to different environmental cues.

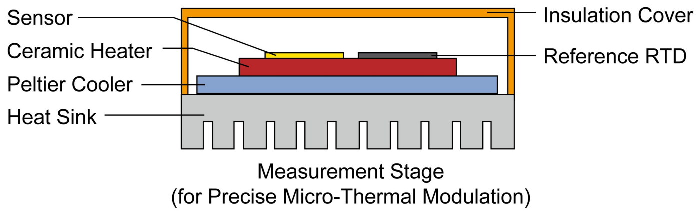

Fig. S30. Custom-built setup for generating subtle thermal variations.

The stage consists of a ceramic heating pad, a Peltier module mounted on a finned aluminum heat sink, and a programmable DC power supply. In this configuration, the ceramic heater provides stable background heating, while the Peltier element enables fine cooling control. Real-time calibration is achieved with a high-resolution commercial reference sensor.

Table S1. Detailed composition of hete-PE and homo-PE.

Hydrogel Matrix			Materials	PEMS (mg)	PSSNa in hete-PE (wt%)
PVA (g)	H_2O (mL)	Gly (g)			
1.00	8.70	0.30	Hete-PE-1	0.20	~0.011%
1.00	8.70	0.30	Hete-PE-2	0.40	~0.023%
1.00	8.70	0.30	Hete-PE-3	0.60	~0.034%
1.00	8.70	0.30	Hete-PE-4	2.00	~0.114%
1.00	8.70	0.30	Hete-PE-5	3.00	~0.171%
1.00	8.70	0.30	Hete-PE-6	4.00	~0.228%
1.00	8.70	0.30	Hete-PE-7	5.00	~0.285%
1.00	8.70	0.30	Hete-PE-8	6.00	~0.342%
1.00	8.70	0.30	Hete-PE-9	20.00	~1.14%
1.00	8.70	0.30	Hete-PE-10	40.00	~2.28%
1.00	8.70	0.30	Hete-PE-11	60.00	~3.42%

Table S2. Summary of state-of-the-art artificial thermal receptor based on ionic conductive materials.

	Materials	Sensitivity (mK)	TCR (%/℃)	B-Index (K)	Range (℃)	Response Time (s)	Mechanical Durability	Thermal Durability	Linearity	Ref.
**Metal**	Ag NFs/Ag NWs	NA	0.03	NA	30-45	NA	NA	30	NA	(49)
	Cr/Cu	NA	0.16	NA	20-120	NA	100	NA	NA	(50)
	Ag NPs/PI	NA	0.17	NA	24-54	NA	NA	NA	NA	(51)
	Ag NFs/Pt NFs	500	0.205	NA	20-60	2	1000	70000 s	NA	(102)
	Ag/PI	NA	0.219	NA	20-60	NA	NA	NA	NA	(52)
	Au	8	0.25	NA	25-50	0.0042	NA	NA	NA	(39)
**Carbons**	CNT-InGaZn	300	0.68	NA	22.4-44.3	11	1000	NA	NA	(53)
	rGO/PU	100	0.8	1066.5	25-46	7	10000	NA	NA	(54)
	rGO	100	0.83	NA	22-70	0.1	10000	NA	R^2>0.99	(55)
	Graphene	NA	1.05	1203	25-100	20	100	NA	NA	(56)
	rGO/PEI	100	1.3	NA	25-45	0.443	500	10/4 month	R^2>0.999	(57)
	rGO/PU	200	1.34	NA	30-80	NA	10000	NA	NA	(58)
	SWCNT	1000	1.435	NA	15-55	NA	NA	100	R^2>0.99	(15)
	Carbon nanofiber	NA	1.52	1855.29	30-85	1.2	100	300	R^2>0.995	(59)
	CNT/XSBR	NA	1.636	5140	30-100	NA	NA	NA	NA	(60)
	GO	200	2.04	1838	26-101	22	NA	NA	NA	(61)
	Carbon fiber	1000	2.44	NA	25-50	0.73	1000	500	NA	(62)
	rGO/PVDF	NA	2.93	NA	20-60	NA	5000	NA	NA	(63)
**2D-Semi-conductor**	BP/LEG on SEBS	NA	0.1736	8106	25-50	NA	NA	10	R^2>0.99	(64)
	MoS_2	NA	0.3	NA	-173-27	NA	NA	NA	NA	(65)
	Monolayer MoS_2	250	2.0	NA	27-85	3.6E-5	NA	1000	NA	(16)
	Li-treated MoS_2	NA	2.0	NA	-173-27	NA	NA	NA	NA	(66)
	MoS_2 thin films	NA	2.9	NA	20-100	12.5	NA	NA	NA	(67)
	Pul-SH/PDA/MoS_2	500	3.3	NA	0-60	6	NA	5	NA	(68)
**Mxene**	MXene/PU Foam	1000	0.22	NA	27-140	NA	NA	NA	NA	(69)
	MXene/TOCNF/PVA	300	1.32	2303.76	20-80	7	20000	40	R^2>0.97	(70)
	MXene/PDA	NA	1.8	NA	20-100	NA	200	NA	R^2>0.99	(71)
	MXene/PU/PVA	NA	5.27	4428	0-80	90	NA	6	NA	(72)
**Conjugated Polymer**	PEDOT:PSS/CNT	NA	0.61	NA	22-48	18	NA	5	NA	(73)
	PVDF/CA/PVA	NA	0.75	NA	27-55	2.1	NA	2000	R^2=0.99	(74)
	PVDF/PANI/Graphene	NA	0.832	NA	20-45	11.05	NA	NA	R^2=0.98	(75)
	PANI NFs/PET	NA	1.0	NA	15-45	NA	1000	NA	R^2=0.998	(17)
	PAM/PEDOT:PSS/ TC/Gly	1000	1.43	NA	20-80	14	200	20	R^2>0.98	(76)
	PAA/PANI/Gly/Fe^{3+}	2700	1.64	NA	40-110	19.5	1000	NA	R^2>0.99	(77)
	PEDOT:PSS/Graphene /PU	100	1.72	NA	30-50	17	NA	20	R^2=0.98	(18)
	PMNB:F4TCNQ/PET	NA	1.92	NA	20-60	9.2	NA	6	NA	(78)
	PEDOT:PSS/PDMS	100	4.2	NA	30-55	NA	NA	10	R^2>0.998	(79)
**SMOCs**	SnO_2/SWCNT/PET	NA	0.97	NA	0-44	NA	NA	NA	NA	(80)
	UCNPs/WO	400	1.53	1229.3	25-95	NA	NA	NA	R^2>0.999	(81)
	V_2O_5/SWCNT	400	1.95	NA	30-80	NA	NA	NA	NA	(82)
	VOx	NA	2.05	2758.62	20-80	NA	NA	NA	NA	(83)
	Cr_2O_3	NA	4.33	3876	25-600	NA	NA	NA	NA	(84)
	Al_2O_3	NA	4.4	3977	250-475	NA	NA	NA	NA	(85)
	VOx	NA	6.5	NA	NA	NA	NA	NA	NA	(86)
	NiO-Ni/PET	1000	9.2	7350	25-70	NA	NA	NA	NA	(87)
	NiO-Ni/PET	1000	9.2	7350	25-70	NA	NA	NA	NA	(14)
**Ionic Liquid Gels**	[EMIM][NTf_2]/TPU	100	1.2	NA	-40-100	NA	NA	5	R^2>0.99	(88)
	[EMIM][NTf_2]/TPU	100	2.1	NA	30-100	NA	NA	5	NA	(89)
	[EMIM][DCA]/ACMO	100	2.7	NA	0-120	NA	NA	10	R^2>0.99	(90)
	EMIM:TFSI/TPU	100	2.73	NA	20-40	8.8	500	5	R^2>0.98	(91)

	[EMIM][NTf2]/ Polyacrylate	50	3.3	NA	25-40	NA	NA	5	R^2>0.99	(92)
	Galinstan/Ionic liquid	640	3.9	NA	25-80	NA	NA	10	NA	(93)
	Li[TFSI]/P(HFA-co-SBMA)	NA	4.15	3895	25-40	NA	NA	10	R^2>0.99	(94)
	LiTFSI/polyNCMA	NA	8.42	7581.05	30-90	NA	200	NA	NA	(26)
**Natural Material**	Silk fibroin/Ca^{2+}	500	1.9	NA	-30-80	NA	NA	20	NA	(95)
	Pectin	10	10.94	9850	0-45	NA	NA	215	NA	(34)
**Hydrogels**	SA/PAAm	500	0.5	NA	22-100	2.02	2000	NA	NA	(96)
	PMMA/Carrageenan	800	0.94	NA	-28-95.3	0.19	700	5	NA	(97)
	Ca-Alg/PAM	NA	1.77	NA	25-50	50.7	1000	NA	R^2>0.99	(98)
	P(AAm/AA)-CS	100	2.9	NA	34-42	86.4	1000	10	R^2=0.998	(99)
	CMCS/SA/SHA/Bn/CS SB	100	4.48	NA	0-135	NA	100	6	R^2>0.998	(100)
	Hete-PE (PSSNa/PVA)	1.2	9.7	8613	25-90	0.3	2000	500	R^2>0.999	This work

Table S3. Summary of key parameters of the several solid materials.

Materials	Thermal Conductivity ($W/m^{-1}\cdot k^{-1}$)
Aluminium	163
Steel	17
Glass	1.46
Rubber	0.20
Wood	0.04

Table S4. Summary of key parameters of the several liquid materials.

Materials	Saturated Vapour Pressure (kPa)	Heat of Vaporization (KJ/mol)	Thermal Conductivity ($W/m^{-1}\cdot k^{-1}$)
Methanol	12.3	35.32	21.35
Ethyl Alcohol	5.8	38.95	18
Acetone	24	29.11	1.98
Ethyl Acetate	10.1	32.28	0.15
Water	2.3	44.23	0.63
N,N-Dimethylformamide	0.5	47.55	0.17

REFERENCES AND NOTES

1. M. Gallio, T. A. Ofstad, L. J. Macpherson, J. W. Wang, C. S. Zuker, The coding of temperature in the *Drosophila* brain. *Cell* **144**, 614–624 (2011).

2. A. Patapoutian, A. M. Peier, G. M. Story, V. Viswanathc, ThermoTRP channels and beyond: Mechanisms of temperature sensation. *Nat. Rev. Neurosci.* **4**, 529–539 (2003).

3. D. D. McKemy, W. M. Neuhausser, D. Julius, Identification of a cold receptor reveals a general role for TRP channels in thermosensation. *Nature* **416**, 52–58 (2002).

4. Y. Huang, J. K. Zhou, P. C. Ke, X. Guo, C. K. Yiu, K. M. Yao, S. Y. Cai, D. F. Li, Y. Zhou, J. Li, T. H. Wong, Y. M. Liu, L. Li, Y. Y. Gao, X. C. Huang, H. Li, J. Y. Li, B. B. Zhang, Z. L. Chen, H. X. Zheng, X. Y. Yang, H. C. Gao, Z. C. Zhao, E. M. Song, H. Wu, Z. K. Wang, Z. Q. Xie, K. N. Zhu, X. G. Yu, A skin-integrated multimodal haptic interface for immersive tactile feedback. *Nat. Electron.* **6**, 1020 (2023).

5. S. Zhao, R. Zhu, Electronic skin with multifunction sensors based on thermosensation. *Adv. Mater.* **29**, 1606151 (2017).

6. T. Someya, M. Amagai, Toward a new generation of smart skins. *Nat. Biotechnol.* **37**, 382–388 (2019).

7. F. Iberite, J. Muheim, O. Akouissi, S. Gallo, G. Rognini, F. Morosato, A. Clerc, M. Kalff, E. Gruppioni, S. Micera, S. Shokur, Restoration of natural thermal sensation in upper-limb amputees. *Science* **380**, 731–735 (2023).

8. A. Chortos, J. Liu, Z. Bao, Pursuing prosthetic electronic skin. *Nat. Mater.* **15**, 937–950 (2016).

9. K. H. Ha, J. Yoo, S. Li, Y. Mao, S. Xu, H. Qi, H. Wu, C. Fan, H. Yuan, J. T. Kim, M. T. Flavin, S. Yoo, P. Shahir, S. Kim, H. Y. Ahn, E. Colgate, Y. Huang, J. A. Rogers, Full freedom-of-motion actuators as advanced haptic interfaces. *Science* **387**, 1383–1390 (2025).

10. R. W. Dykes, Coding of steady and transient temperatures by cutaneous "Cold" fibers serving the hand of monkeys. *Brain Res.* **98**, 485–500 (1975).

11. E. O. Gracheva, N. T. Ingolia, Y. M. Kelly, J. F. Cordero-Morales, G. Hollopeter, A. T. Chesler, E. E. Sanchez, J. C. Perez, J. S. Weissman, D. Julius, Molecular basis of infrared detection by snakes. *Nature* **464**, 1006–1011 (2010).

12. B. R. Brown, Sensing temperature without ion channels. *Nature* **421**, 495 (2003).

13. Z. Gong, W. Di, Y. Jiang, Z. Dong, Z. Yang, H. Ye, H. Zhang, H. Liu, Z. Wei, Z. Tu, D. Li, J. Xiang, X. Ding, D. Zhang, H. Chen, Flexible calorimetric flow sensor with unprecedented sensitivity and directional resolution for multiple flight parameter detection. *Nat. Commun.* **15**, 3091 (2024).

14. J. Shin, B. Jeong, J. Kim, V. B. Nam, Y. Yoon, J. Jung, S. Hong, H. Lee, H. Eom, J. Yeo, J. Choi, D. Lee, S. H. Ko, Sensitive wearable temperature sensor with seamless monolithic integration. *Adv. Mater.* **32**, 1905527.15 (2020).

15. C. Zhu, A. Chortos, Y. Wang, R. Pfattner, T. Lei, A. C. Hinckley, I. Pochorovski, X. Yan, J. W. F. To, J. Y. Oh, J. B. H. Tok, Z. Bao, B. Murmann, Stretchable temperature-sensing circuits with strain suppression based on carbon nanotube transistors. *Nat. Electron.* **1**, 183–190 (2018).

16. A. Daus, M. Jaikissoon, A. I. Khan, A. Kumar, R. W. Grady, K. C. Saraswat, E. Pop, Fast-response flexible temperature sensors with atomically thin molybdenum disulfide. *Nano Lett.* **22**, 6135–6140 (2022).

17. S. Y. Hong, Y. H. Lee, H. Park, S. W. Jin, Y. R. Jeong, J. Yun, I. You, G. Zi, J. S. Ha, Stretchable active matrix temperature sensor array of polyaniline nanofibers for electronic skin. *Adv. Mater.* **28**, 930–935 (2016).

18. W. Fan, T. Liu, F. Wu, S. Wang, S. Ge, Y. Li, J. Liu, H. Ye, R. Lei, C. Wang, Q. Che, Y. Li, An antisweat interference and highly sensitive temperature sensor based on poly(3,4-

ethylenedioxythiophene)–poly(styrenesulfonate) fiber coated with polyurethane/graphene for real-time monitoring of body temperature. *ACS Nano* **17**, 21073–21082 (2023).

19. J. Jeon, H. B. Lee, Z. Bao, Flexible wireless temperature sensors based on Ni microparticle-filled binary polymer composites. *Adv. Mater.* **25**, 850–855 (2013).

20. T. Yokota, Y. Inoue, Y. Terakawa, J. Reeder, M. Kaltenbrunner, T. Ware, K. Yang, K. Mabuchi, T. Murakawa, M. Sekino, W. Voit, T. Sekitani, T. Someya, Ultraflexible, large-area, physiological temperature sensors for multipoint measurements. *Proc. Natl. Acad. Sci. U.S.A.* **112**, 14533–14538 (2015).

21. C. H. Yang, Z. G. Suo, Hydrogel ionotronics. *Nat. Rev. Mater.* **3**, 125–142 (2018).

22. Y. Li, N. Bai, Y. Chang, Z. Liu, J. Liu, X. Li, W. Yang, H. Niu, W. Wang, L. Wang, W. Zhu, D. Chen, T. Pan, C. Guo, G. Shen, Flexible iontronic sensing. *Chem. Soc. Rev.* **54**, 4651–4700 (2025).

23. Y. Wang, K. Jia, Z. Suo, Non-faradaic junction sensing. *Nat. Rev. Mater.* **10**, 176–190 (2025).

24. Y. Xiong, J. Han, Y. Wang, Z. Wang, Q. Sun, Emerging iontronic sensing: Materials, mechanisms, and applications. *Research* **2022**, 9867378 (2022).

25. T. H. Kim, Z. Zhou, Y. S. Choi, V. Costanza, L. Wang, J. H. Bahng, N. J. Higdon, Y. Yun, H. Kang, S. Kim, C. Daraio, Flexible biomimetic block copolymer composite for temperature and long-wave infrared sensing. *Sci. Adv.* **9**, 0423 (2023).

26. P. Yao, Q. Bao, Y. Yao, M. Xiao, Z. Xu, J. Yang, W. Liu, Environmentally stable, robust, adhesive, and conductive supramolecular deep eutectic gels as ultrasensitive flexible temperature sensor. *Adv. Mater.* **35**, e2300114 (2023).

27. Y. Wang, K. Jia, S. Zhang, H. J. Kim, Y. Bai, R. C. Hayward, Z. Suo, Temperature sensing using junctions between mobile ions and mobile electrons. *Proc. Natl. Acad. Sci. U.S.A.* **199**, e2117962119 (2022).

28. R. Wang, Y. Sun, F. Zhang, M. Song, D. Tian, H. Li, Temperature-sensitive artificial channels through pillar[5]arene-based host–guest interactions. *Angew. Chem. Int. Ed.* **56**, 5294–5298 (2017).

29. Z. Song, F. Chen, M. Martinez-Ibañez, W. Feng, M. Forsyth, Z. Zhou, M. Armand, H. Zhang, A reflection on polymer electrolytes for solid-state lithium metal batteries. *Nat. Commun.* **14**, 4884 (2023).

30. H. Gao, N. S. Grundish, Y. Zhao, A. Zhou, J. B. Goodenough, Formation of stable interphase of polymer-in-salt electrolyte in all-solid-state lithium batteries. *Energy Mater. Adv.* **2021**, 1932952 (2021).

31. A. K. Jonscher, Dielectric relaxation in solids. *J. Phys. D* **32**, 57–70 (1999).

32. C. Gainaru, E. W. Stacy, V. Bocharova, M. Gobet, A. P. Holt, T. Saito, S. Greenbaum, A. P. Sokolov, Mechanism of conductivity relaxation in liquid and polymeric electrolytes: Direct link between conductivity and diffusivity. *J. Phys. Chem. B* **120**, 11074–11083 (2016).

33. B. A. Mei, O. Munteshari, J. Lau, B. Dunn, L. Pilon, Physical interpretations of nyquist plots for EDLC rlectrodes and devices. *J. Phys. Chem. C* **122**, 194–206 (2017).

34. R. Di Giacomo, L. Bonanomi, V. Costanza, B. Maresca, C. Daraio, Biomimetic temperature-sensing layer for artificial skins. *Sci. Robot.* **2**, 9251 (2017).

35. R. Di Giacomo, C. Daraio, B. Maresca, Plant nanobionic materials with a giant temperature response mediated by pectin-Ca^{2+}. *Proc. Natl. Acad. Sci. U.S.A.* **112**, 4541–4545 (2015).

36. H. Yin, F. Liu, T. Abdiryim, J. Chen, X. Liu, Sodium carboxymethyl cellulose and MXene reinforced multifunctional conductive hydrogels for multimodal sensors and flexible supercapacitors. *Carbohyd. Polym.* **327**, 121677 (2024).

37. Y. Zhou, C. Yu, X. Zhang, Y. Zheng, B. Wang, Y. Bao, G. Shan, H. Wang, P. Pan, Ultrasensitive ionic conductors with tunable resistance switching temperature enabled by phase transformation of polymer cocrystals. *Adv. Mater.* **36**, e2309568 (2024).

38. C. Zhou, X. Song, W. Xia, S. Liu, Z. Wu, H. Chen, Recyclable multifunctional ion conductive elastomers for strain/temperature sensors and bioelectrodes. *Chem. Eng. J.* **489**, 151433 (2024).

39. R. C. Webb, A. P. Bonifas, A. Behnaz, Y. Zhang, K. J. Yu, H. Cheng, M. Shi, Z. Bian, Z. Liu, Y. Kim, W. Yeo, J. S. Park, J. Song, Y. Li, Y. Huang, A. M. Gorbach, J. A. Rogers, Ultrathin conformal devices for precise and continuous thermal characterization of human skin. *Nat. Mater.* **12**, 938–944 (2013).

40. Y. Luo, M. R. Abidian, J.-H. Ahn, D. Akinwande, A. M. Andrews, M. Antonietti, Z. Bao, M. Berggren, C. A. Berkey, C. J. Bettinger, J. Chen, P. Chen, W. Cheng, X. Cheng, S.-J. Choi, A. Chortos, C. Dagdeviren, R. H. Dauskardt, C.-a. Di, M. D. Dickey, X. Duan, A. Facchetti, Z. Fan, Y. Fang, J. Feng, X. Feng, H. Gao, W. Gao, X. Gong, C. F. Guo, X. Guo, M. C. Hartel, Z. He, J. S. Ho, Y. Hu, Q. Huang, Y. Huang, F. Huo, M. M. Hussain, A. Javey, U. Jeong, C. Jiang, X. Jiang, J. Kang, D. Karnaushenko, A. Khademhosseini, D.-H. Kim, I.-D. Kim, D. Kireev, L. Kong, C. Lee, N.-E. Lee, P. S. Lee, T.-W. Lee, F. Li, J. Li, C. Liang, C. T. Lim, Y. Lin, D. J. Lipomi, J. Liu, K. Liu, N. Liu, R. Liu, Y. Liu, Y. Liu, Z. Liu, Z. Liu, X. J. Loh, N. Lu, Z. Lv, S. Magdassi, G. G. Malliaras, N. Matsuhisa, A. Nathan, S. Niu, J. Pan, C. Pang, Q. Pei, H. Peng, D. Qi, H. Ren, J. A. Rogers, A. Rowe, O. G. Schmidt, T. Sekitani, D.-G. Seo, G. Shen, X. Sheng, Q. Shi, T. Someya, Y. Song, E. Stavrinidou, M. Su, X. Sun, K. Takei, X.-M. Tao, B. C. K. Tee, A. V.-Y. Thean, T. Q. Trung, C. Wan, H. Wang, J. Wang, M. Wang, S. Wang, T. Wang, Z. L. Wang, P. S. Weiss, H. Wen, S. Xu, T. Xu, H. Yan, X. Yan, H. Yang, L. Yang, S. Yang, L. Yin, C. Yu, G. Yu, J. Yu, S.-H. Yu, X. Yu, E. Zamburg, H. Zhang, X. Zhang, X. Zhang, X. Zhang, Y. Zhang, Y. Zhang, S. Zhao, X. Zhao, Y. Zheng, Y.-Q. Zheng, Z. Zheng, T. Zhou, B. Zhu, M. Zhu, R. Zhu, Y. Zhu, Y. Zhu, G. Zou, X. Chen, Technology roadmap for flexible sensors. *ACS Nano* **17**, 5211–5295 (2023).

41. Y. Yuan, B. Zhong, X. Qin, H. Xu, Z. Li, L. Li, X. Wang, W. Zhang, Z. Lou, Y. Fan, L. Wang, An epidermal serine sensing system for skin healthcare. *Nat. Commun.* **16**, 2681 (2025).

42. J. Vriens, B. Nilius, T. Voets, Peripheral thermosensation in mammals. *Nat. Rev. Neurosci.* **15**, 573–589 (2014).

43. K. Nakamura, S. F. Morrison, A thermosensory pathway that controls body temperature. *Nat Neurosci.* **11**, 62–71 (2008).

44. J. Yang, R. Bai, B. Chen, Z. Suo, Hydrogel adhesion: A supramolecular synergy of chemistry, topology, and mechanics. *Adv. Funct. Mater.* **30**, 1901693 (2020).

45. X. Liu, X. Ji, R. Zhu, J. Gu, J. Liang, A microphase-separated design toward an all-round ionic hydrogel with discriminable and anti-disturbance multisensory functions. *Adv. Mater.* **36**, e2309508 (2024).

46. Z. Li, H. Xu, Y. Zheng, L. Liu, L. Li, Z. Lou, L. Wang, A reconfigurable heterostructure transistor array for monocular 3D parallax reconstruction. *Nat. Electron.* **8**, 46–55 (2025).

47. L. Li, H. Xu, Z. Li, B. Zhong, Z. Lou, L. Wang, 3D heterogeneous sensing system for multimode parrallel signal no-spatiotemporal misalignment recognition. *Adv. Mater.* **37**, e2414054 (2025).

48. J. Xu, L. Peng, S. Yuan, S. Li, H. Zhu, L. Fu, T. Zhang, T. Li, Advanced optical-thermal integrated flexible tactile sensor for high-fine recognition of liquid property in non-contact mode. *Adv. Funct. Mater.* **34**, 2410885 (2024).

49. B. W. An, S. Heo, S. Ji, F. Bien, J.-U. Park, Transparent and flexible fingerprint sensor array with multiplexed detection of tactile pressure and skin temperature. *Nat. Commun.* **9**, 2458 (2018).

50. C. Yu, Z. Wang, H. Yu, H. Jiang, A stretchable temperature sensor based on elastically buckled thin film devices on elastomeric substrates. *Appl. Phys. Lett.* **95**, 141912 (2009).

51. Z. Zou, C. Zhu, Y. Li, X. Lei, W. Zhang, J. Xiao, Rehealable, fully recyclable, and malleable electronic skin enabled by dynamic covalent thermoset nanocomposite. *Sci. Adv.* **4**, 0508 (2018).

52. M. D. Dankoco, G. Y. Tesfay, E. Benevent, M. Bendahan, Temperature sensor realized by inkjet printing process on flexible substrate. *Mater. Sci. Eng. B* **205**, 1–5 (2016).

53. W. Honda, S. Harada, S. Ishida, T. Arie, S. Akita, K. Takei, High-performance, mechanically flexible, and vertically integrated 3D carbon nanotube and ingazno complementary circuits with a temperature sensor. *Adv. Mater.* **27**, 4674–4680 (2015).

54. T. Q. Trung, T. M. L. Dang, S. Ramasundaram, P. T. Toi, S. Y. Park, N.-E. Lee, A stretchable strain-insensitive temperature sensor based on free-standing elastomeric composite fibers for on-body monitoring of skin temperature. *ACS Appl. Mater. Interfaces* **11**, 2317–2327 (2019).

55. G. Y. Bae, J. T. Han, G. Lee, S. Lee, S. W. Kim, S. Park, J. Kwon, S. Jung, K. Cho, Pressure/temperature sensing bimodal electronic skin with stimulus discriminability and linear sensitivity. *Adv. Mater.* **30**, e1803388 (2018).

56. C. Yan, J. Wang, P. S. Lee, Stretchable graphene thermistor with tunable thermal index. *ACS Nano* **9**, 2130–2137 (2015).

57. Q. Liu, H. Tai, Z. Yuan, Y. Zhou, Y. Su, Y. Jiang, A high-performances flexible temperature sensor composed of polyethyleneimine/reduced graphene oxide bilayer for real-time monitoring. *Adv. Mat. Technol.* **4**, 1800594 (2019).

58. T. Q. Trung, S. Ramasundaram, B.-U. Hwang, N.-E. Lee, An all-elastomeric transparent and stretchable temperature sensor for body-attachable wearable electronics. *Adv. Mater.* **28**, 502–509 (2016).

59. J.-H. Lee, H. Chen, E. Kim, H. Zhang, K. Wu, H. Zhang, X. Shen, Q. Zheng, J. Yang, S. Jeon, J.-K. Kim, Flexible temperature sensors made of aligned electrospun carbon nanofiber films with outstanding sensitivity and selectivity towards temperature. *Mat. Horiz.* **8**, 1488–1498 (2021).

60. M. Lin, Z. Zheng, L. Yang, M. Luo, L. Fu, B. Lin, C. Xu, A. High-Performance, A high-performance, sensitive, wearable multifunctional sensor based on rubber/Cnt for human motion and skin temperature detection. *Adv. Mater.* **34**, e2107309 (2022).

61. J. Wu, W. Huang, Y. Liang, Z. Wu, B. Zhong, Z. Zhou, J. Ye, K. Tao, Y. Zhou, X. Xie, Self-calibrated, sensitive, and flexible temperature sensor based on 3D chemically modified graphene hydrogel. *Adv. Electron. Mater.* **7**, 2001084 (2021).

62. J.-H. Lee, E. Kim, H. Zhang, H. Chen, H. Venkatesan, K.-Y. Chan, J. Yang, X. Shen, J. Yang, S. Jeon, J.-K. Kim, Rational design of all resistive multifunctional sensors with stimulus discriminability. *Adv. Funct. Mater.* **32**, 2107570 (2022).

63. J. Park, M. Kim, Y. Lee, H. S. Lee, H. Ko, Fingertip skin–inspired microstructured ferroelectric skins discriminate static/dynamic pressure and temperature stimuli. *Sci. Adv.* **1**, 1500661 (2015).

64. A. Chhetry, S. Sharma, S. C. Barman, H. Yoon, S. Ko, C. Park, S. Yoon, H. Kim, J. Y. Park, Black phosphorus@laser-engraved graphene heterostructure-based temperature–strain hybridized sensor for electronic-skin applications. *Adv. Funct. Mater.* **31**, 2007661 (2021).

65. A. I. Khan, P. Khakbaz, K. A. Brenner, K. K. H. Smithe, M. J. Mleczko, D. Esseni, E. Pop, Large temperature coefficient of resistance in atomically thin two-dimensional semiconductors. *Appl. Phys. Lett.* **116**, 203105 (2020).

66. J. S. Kim, J. Kim, J. Zhao, S. Kim, J. H. Lee, Y. Jin, H. Choi, B. H. Moon, J. J. Bae, Y. H. Lee, S. C. Lim, Electrical transport properties of polymorphic MoS_2. *ACS Nano* **10**, 7500–7506 (2016).

67. A. Goswami, P. Dhandaria, S. Pal, R. McGee, F. Khan, Ž. Antić, R. Gaikwad, K. Prashanthi, T. Thundat, Effect of interface on mid-infrared photothermal response of mos_2 thin film grown by pulsed laser deposition. *Nano Res.* **10**, 3571–3584 (2017).

68. S. Roy, K. A. Deo, H. P. Lee, J. Soukar, M. Namkoong, L. Tian, A. Jaiswal, A. K. Gaharwar, 3D printed electronic skin for strain, pressure and temperature sensing. *Adv. Funct. Mater.* **34**, 2313575 (2024).

69. V. Adepu, V. Mattela, P. Sahatiya, A remarkably ultra-sensitive large area matrix of mxene based multifunctional physical sensors (pressure, strain, and temperature) for mimicking human skin. *J. Mater. Chem. B* **9**, 4523–4534 (2021).

70. S. Hao, Q. Fu, L. Meng, F. Xu, J. Yang, A biomimetic laminated strategy enabled strain-interference free and durable flexible thermistor electronics. *Nat. Commun.* **13**, 6472 (2022).

71. J. Luo, S. Gao, H. Luo, L. Wang, X. Huang, Z. Guo, X. Lai, L. Lin, R. K. Y. Li, J. Gao, Superhydrophobic and breathable smart mxene-based textile for multifunctional wearable sensing electronics. *Chem. Eng. J.* **406**, 126898 (2021).

72. H. Liu, C. Du, L. Liao, H. Zhang, H. Zhou, W. Zhou, T. Ren, Z. Sun, Y. Lu, Z. Nie, F. Xu, J. Zhu, W. Huang, Approaching intrinsic dynamics of mxenes hybrid hydrogel for 3D printed multimodal intelligent devices with ultrahigh superelasticity and temperature sensitivity. *Nat. Commun.* **13**, 3420 (2022).

73. W. Honda, S. Harada, T. Arie, S. Akita, K. Takei, Wearable, human-interactive, health-monitoring, wireless devices fabricated by macroscale printing techniques. *Adv. Funct. Mater.* **24**, 3299–3304 (2014).

74. O. Yue, X. Wang, X. Liu, M. Hou, M. Zheng, Y. Wang, B. Cui, Spider-web and ant-tentacle doubly bio-inspired multifunctional self-powered electronic skin with hierarchical nanostructure. *Adv. Sci.* **8**, 2004377 (2021).

75. C. Zhang, W. Wei, Y. Li, X. Li, Y. Liu, X. Wang, S. Chen, Sweat and deformation-resistance graphite/PVDF/PANI-based temperature sensor for real-time body temperature monitoring. *Adv. Mat. Technol.* **9**, 2400149 (2024).

76. S. Hao, R. Dai, Q. Fu, Y. Wang, X. Zhang, H. Li, X. Liu, J. Yang, A robust and adhesive hydrogel enables interfacial coupling for continuous temperature monitoring. *Adv. Funct. Mater.* **33**, 2302840 (2023).

77. G. Ge, Y. Lu, X. Qu, W. Zhao, Y. Ren, W. Wang, Q. Wang, W. Huang, X. Dong, Muscle-inspired self-healing hydrogels for strain and temperature sensor. *ACS Nano* **14**, 218–228 (2020).

78. J. H. L. Ngai, J. Polena, D. Afzal, X. Gao, M. Kapadia, Y. Li, Green solvent-processed hemi-isoindigo polymers for stable temperature sensors. *Adv. Funct. Mater.* **32**, 2110995 (2022).

79. Y. Yu, S. Peng, P. Blanloeuil, S. Wu, C. H. Wang, Wearable temperature sensors with enhanced sensitivity by engineering microcrack morphology in PEDOT:PSS–PDMS sensors. *ACS Appl. Mater. Interfaces* **12**, 36578–36588 (2020).

80. K. Xu, Y. Lu, T. Yamaguchi, T. Arie, S. Akita, K. Takei, Highly precise multifunctional thermal management-based flexible sensing sheets. *ACS Nano* **13**, 14348–14356 (2019).

81. W. Zhang, X. Huang, W. Liu, Z. Gao, L. Zhong, Y. Qin, B. Li, J. Li, Semiconductor plasmon enhanced upconversion toward a flexible temperature sensor. *ACS Appl. Mater. Interfaces* **15**, 4469–4476 (2023).

82. J. Zhao, Y. Zhang, Y. Huang, J. Xie, X. Zhao, C. Li, J. Qu, Q. Zhang, J. Sun, B. He, Q. Li, C. Lu, X. Xu, W. Lu, L. Li, Y. Yao, 3D printing fiber electrodes for an all-fiber integrated electronic device via hybridization of an asymmetric supercapacitor and a temperature sensor. *Adv. Sci.* **5**, 1801114 (2018).

83. J. Dai, X. Wang, S. He, Y. Huang, X. Yi, Low temperature fabrication of vox thin films for uncooled ir detectors by direct current reactive magnetron sputtering method. *Infrared Phys. Technol.* **51**, 287–291 (2008).

84. J. Park, Microstructural and electrical properties of $Y_{0.2}Al_{0.1}Mn_{0.27-X}Fe_{0.16}Ni_{0.27-X}(Cr_{2x})O_y$ for NTC thermistors. *Ceram. Int.* **41**, 6386–6390 (2015).

85. J. Xia, Q. Zhao, A. Chang, B. Zhang, Synthesis and properties of $Mn_{1.05-Y}Co_{1.95-X-Z-W}Ni_xMg_yAl_zFe_w$O4 NTC ceramic by co-precipitation method. *J. Alloys Compd.* **646**, 249–256 (2015).

86. C. Ma, Y. Liu, Y. Lu, H. Qian, Preparation and electrical properties of $Ni_{0.6}Mn_{2.4-X}Ti_{x}o_{4}$ NTC ceramics. *J. Alloys Compd.* **650**, 931 (2015).

87. B. Wang, J. Lai, H. Li, H. Hu, S. Chen, Nanostructured vanadium oxide thin film with high Tcr at room temperature for microbolometer. *Infrared Phys. Technol.* **57**, 8–13 (2013).

88. N. Jiang, X. Chang, D. Hu, L. Chen, Y. Wang, J. Chen, Y. Zhu, Flexible, transparent, and antibacterial ionogels toward highly sensitive strain and temperature sensors. *Chem. Eng. J.* **424**, 130418 (2021).

89. Y. Xu, L. Chen, J. Chen, X. Chang, Y. Zhu, Flexible and transparent pressure/temperature sensors based on ionogels with bioinspired interlocked microstructures. *ACS Appl. Mater. Interfaces* **14**, 2122–2131 (2022).

90. J. Tie, Z. Mao, L. Zhang, Y. Zhong, H. Xu, Strong and ultratough ionogel enabled by ingenious combined ionic liquids induced microphase separation. *Adv. Funct. Mater.* **33**, 2307367 (2023).

91. F. Li, H. Xue, X. Lin, C. Zhao, J. Li, H. Zhao, T. Zhang, Ionic gel based multifunctional sensor for body temperature monitoring and joint motion detection. *Adv. Mat. Technol.* **8**, 2300297 (2023).

92. H. Wang, Y. Mao, D. Ji, L. Wang, L. Wang, J. Chen, X. Chang, Y. Zhu, Transparent, self-adhesive, highly environmental stable, and water-resistant ionogel enabled reliable strain/temperature sensors and underwater communicators. *Chem. Eng. J.* **471**, 144674 (2023).

93. H. Ota, K. Chen, Y. Lin, D. Kiriya, H. Shiraki, Z. Yu, T.-J. Ha, A. Javey, Highly deformable liquid-state heterojunction sensors. *Nat. Commun.* **5**, 5032 (2014).

94. Y. Zheng, J. Wang, T. Cui, M. Zhang, L. Yang, Y. Hu, Z. Gui, Transparent ionogel balancing rigidity and flexibility with prolonged stability for ultra-high sensitivity temperature sensing. *Chem. Eng. J.* **494**, 152695 (2024).

95. J. Liu, Q. Chen, Q. Liu, B. Zhao, S. Ling, J. Yao, Z. Shao, X. Chen, Intelligent silk fibroin ionotronic skin for temperature sensing. *Adv. Mat. Technol.* **5**, 2000430 (2020).

96. J. Zhang, K. Yan, J. Huang, X. Sun, J. Li, Y. Cheng, Y. Sun, Y. Shi, L. Pan, Mechanically robust, flexible, fast responding temperature sensor and high-resolution array with ionically conductive double cross-linked hydrogel. *Adv. Funct. Mater.* **34**, 2314433 (2024).

97. Z. Wu, H. Ding, K. Tao, Y. Wei, X. Gui, W. Shi, X. Xie, J. Wu, Ultrasensitive, stretchable, and fast-response temperature sensors based on hydrogel films for wearable applications. *ACS Appl. Mater. Interfaces* **13**, 21854–21864 (2021).

98. W. Wang, D. Yao, H. Wang, Q. Ding, Y. Luo, H. Ding, J. Yu, H. Zhang, K. Tao, S. Zhang, F. Huo, J. Wu, A. Breathable, A breathable, stretchable, and self-calibrated multimodal electronic skin based on hydrogel microstructures for wireless wearables. *Adv. Funct. Mater.* **34**, 2316339 (2024).

99. Y. Li, D. Li, J. Wang, T. Ye, Q. Li, L. Li, R. Gao, Y. Wang, J. Ren, F. Li, J. Lu, E. He, Y. Jiao, L. Wang, Y. Zhang, A temperature-sensing hydrogel coating on the medical catheter. *Adv. Funct. Mater.* **34**, 2310260 (2024).

100. B. Zhang, L. Rong, Z. Zhou, W. Yuan, Ultra-stretchable, high-adhesive, self-healable and remoldable hydrogel sensor with dynamic multi-interactions for multiscale motion detection, braille transmission and temperature monitoring. *Chem. Eng. J.* **462**, 142305 (2023).

101. I. Dellatolas, M. Bantawa, B. Damerau, M. Guo, T. Divoux, E. Del Gado, I. Bischofberger, Local mechanism governs global reinforcement of nanofiller-hydrogel composites. *ACS Nano* **17**, 20939–20948 (2023).

102. J. Huang, Z. Xu, W. Qiu, F. Chen, Z. Meng, C. Hou, W. Guo, X. Y. Liu, Stretchable and heat-resistant protein-based electronic skin for human thermoregulation. *Adv. Funct. Mater.* **30**, 1910547 (2020).
